# Conformational Analysis and Organocatalytic Activity of Helical Stapled Peptides Containing α-Carbocyclic α,α-Disubstituted α-Amino Acids

**DOI:** 10.3390/molecules29184340

**Published:** 2024-09-12

**Authors:** Akihiro Iyoshi, Atsushi Ueda, Tomohiro Umeno, Takuma Kato, Kazuhiro Hirayama, Mitsunobu Doi, Masakazu Tanaka

**Affiliations:** 1 Graduate School of Biomedical Sciences, Nagasaki University, 1-14 Bunkyo-machi, Nagasaki 852-8521, Japan; bb55723002@ms.nagasaki-u.ac.jp (A.I.); umeno@koto.kpu-m.ac.jp (T.U.);; 2 Faculty of Pharmacy, Osaka Medical and Pharmaceutical University, Osaka 569-1094, Japan; takuma.kato@ompu.ac.jp (T.K.); mitsunobu.doi@ompu.ac.jp (M.D.)

**Keywords:** peptide, α-helix, organocatalyst, epoxidation

## Abstract

Conformational freedom-restricted peptides, such as stapled peptides, play a crucial role in the advancement of functional peptide development. We synthesized stapled octapeptides using α-carbocyclic α,α-disubstituted α-amino acids, particularly 3-allyloxy-1-aminocyclopentane-1-carboxylic acid, as the crosslink motifs. The organocatalytic capabilities of the synthesized stapled peptides were assessed in an asymmetric nucleophilic epoxidation reaction because the catalytic activities are known to be proportional to α-helicity. Despite incorporating side-chain crosslinks, the enantioselectivities of the epoxidation reaction catalyzed by stapled octapeptides were found to be comparable to those obtained using unstapled peptides. Interestingly, the stapled peptides using α-carbocyclic α,α-disubstituted α-amino acids demonstrated higher reactivities and stereoselectivities (up to 99% ee) compared to stapled peptides derived from (*S*)-α-(4-pentenyl)alanine, a commonly used motif for stapled peptides. These differences could be attributed to the increased α-helicity of the former stapled peptide in contrast to the latter, as evidenced by the X-ray crystallographic structures of their *N*-*tert*-butoxycarbonyl derivatives.

## 1. Introduction

Conformationally restricted peptides typically adopt specific secondary structures, such as α-helices, β-turns, or β-sheets, which are advantageous for the development of peptide foldamers intended for use as organocatalysts [1,2,3,4]. For example, helical peptides demonstrate efficacy as organocatalysts for asymmetric reactions such as epoxidation [5,6,7,8,9,10,11,12,13,14,15,16,17], Michael addition [18,19,20,21,22,23,24], and transphosphorylation [25,26]. Similarly, β-turn peptides can serve as organocatalysts in asymmetric reactions, including kinetic resolution [27], conjugate addition [28,29], and epoxidation [30,31]. Hence, synthesizing peptides with stable secondary structures holds paramount importance in the field of peptide catalyst development. The Juliá–Colonna epoxidation is an asymmetric nucleophilic epoxidation of chalcones catalyzed by poly(L-α-amino acids) under triphasic and biphasic conditions [5,6,7]. The stereoselectivity observed in the Juliá–Colonna epoxidation correlates directly with the α-helical content of the catalysts [6,9,11]. Incorporating α,α-disubstituted α-amino acids (dAAs) into peptides not only enhances the helicity of the peptide secondary structure but also augments their activity [32,33,34,35,36,37,38,39,40]. Takagi and Ohkata et al. developed oligo-L-leucine helical peptide catalysts containing 2-aminoisobutyric acid (Aib), rendering them soluble in organic solvents [10]. Consequently, in prior work, we achieved the enantioselective Juliá–Colonna-type epoxidation of α,β-unsaturated ketones catalyzed by 5 mol % L-leucine-based nonapeptides incorporating α-carbocyclic dAA, (1*S*,3*S*)-1-amino-3-methoxycyclopentane-1-carboxylic acid [Ac_5_c^OM^], yielding 96–98% ee of epoxides [14]. Additionally, introducing a crosslink on the peptide side-chain presents another option to stabilize secondary structures. All-hydrocarbon stapling, as developed by Verdine et al., can enhance the α-helicity of peptides through ring-closing metathesis reactions involving (*S*)-α-(4-pentenyl)alanine residues at the *i*, *i* + 4 or *i*, *i* + 7 positions [41]. Hence, stapled peptides offer potential as organocatalysts. For example, Demizu et al. illustrated that a hydrocarbon-stapled peptide linking *O*-allyl-L-homoserine residues at the *i*, *i* + 4 position could enhance the enantioselectivity of the Juliá–Colonna-type epoxidation reaction [15,16]. Moreover, a side-chain tethered α-helical peptide linking *cis*-4-allyloxy-L-proline and *O*-allyl-L-serine at the *i*, *i* + 1 position served as an organocatalyst for the Michael addition of 1-methylindole to the α,β-unsaturated aldehyde, with improved conversion observed following the introduction of the side-chain staple [42,43]. These findings motivated us to explore the development of helical peptides containing α-carbocyclic dAAs incorporating side-chain hydrocarbon stapling motifs [44,45]. Herein, we report the synthesis of α-helical peptides containing α-carbocyclic dAAs, specifically 3-allyloxy-1-aminocyclopentane-1-carboxylic acids (Ac_5_c^OAll^), along with their secondary structures and organocatalytic activities in the asymmetric epoxidation of α,β-unsaturated ketones. This report also aimed to compare the structural and catalytic features of the stapled peptides incorporating Ac_5_c^OAll^ and (*S*)-α-(4-pentenyl)alanine, a commonly used peptide stapling motif [46,47].

## 2. Results and Discussion

**Scheme 1:**
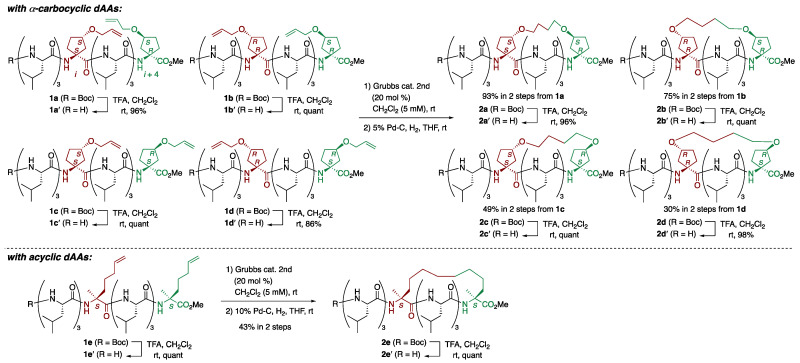
Synthesis of the peptide catalysts **1a**′–**1e**′ and **2a**′–**2e**′ with hydrocarbon stapling at *i* (brown) and *i* + 4 (green) positions.

Because N-terminal-free peptide catalysts demonstrate higher catalytic activity than N-protected peptide catalysts in the nucleophilic epoxidation reactions [14], we chose N-terminal-free peptides **1a**′–**1e**′ and **2a**′–**2e**′ as catalysts in this study (Scheme 1). The synthesis of unstapled peptide catalysts **1a**′–**1d**′ involved the *tert*-butoxycarbonyl (Boc) deprotection of previously reported octapeptides **1a**–**1d** under acidic conditions [45]. The ring-closing metathesis reaction of peptide **1a**, utilizing a second-generation Grubbs catalyst, followed by hydrogenation of the resultant double bond, yielded stapled peptide **2a** in 93% yield in two steps. Similarly, stapled peptides **2b**–**2d**, characterized by different stereochemical patterns of dAAs, were also synthesized from **1b** to **1d**, in 75–30% yields over two steps, with the recovery of starting materials **1b**–**1d** (24–58%) in the first step. Finally, the stapled peptide catalysts **2a**′–**2d**′ were obtained after Boc deprotection in yields ranging from 96% to quantitative yields. Stapled peptide **2e,** derived from **1e** [45], which contains acyclic dAAs of (*S*)-α-(4-pentenyl)alanine, was synthesized in a manner similar to that described for the synthesis of **2a**, in 43% yield over two steps. Boc deprotection of peptides **1e** and **2e** yielded peptide catalysts **1e**′ and **2e**′, respectively, in quantitative yield.

Next, the synthesized peptide catalysts, denoted as **1a**′–**1e**′ and **2a**′–**2e**′, underwent assessment in the asymmetric epoxidation of chalcone **3a** (Table 1). The reaction utilized a 5 mol % peptide catalyst with a 1.1 equivalent of urea hydrogen peroxide and 5.6 equivalent of DBU. All reactions, except for those involving peptide **2e**′, progressed quantitatively, yielding *trans*-epoxide **4a**. Enantioselectivities of the reactions were comparable despite the presence or absence of the side-chain crosslink in the catalysts (entries 2, 4, 6, 8, 10 vs. entries 1, 3, 5, 7, 9). Notably, reactions catalyzed by the acyclic dAA-containing peptide **1e**′ and its stapled derivative **2e**′ resulted in reduced enantiomeric excesses of 89% ee and 90% ee, respectively (entries 9 and 10). These results indicate that the introduction of dAAs to peptide catalysts significantly influences the stereoselectivity compared to the introduction of the side-chain crosslink. Moreover, peptides containing α-carbocyclic dAAs demonstrate greater suitability than peptides incorporating acyclic dAAs for this reaction.

**Figure 1:**
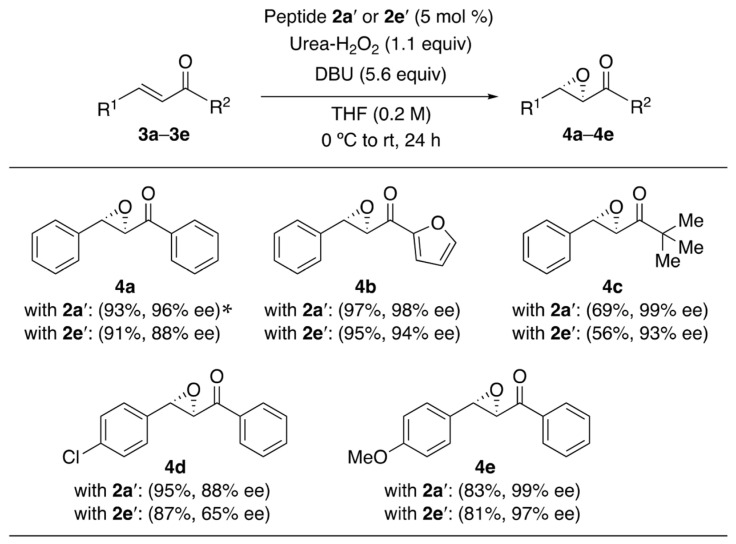
Comparison of the catalytic activities of peptides **2a**′ and **2e**′ with various α,β-unsaturated ketones. Isolated yields are shown. Ee was determined by HPLC. * Conversion was >99% determined by ^1^H NMR analysis.

To investigate the impact of α-carbocyclic dAAs in comparison to acyclic dAAs on the catalytic activities, various α,β-unsaturated ketones were subjected to asymmetric epoxidation reactions in the presence of catalysts **2a**′ or **2e**′ (Figure 1). Substituting the α′-phenyl group with a 2-furyl group in the substrate resulted in a slight enhancement of enantioselectivities to 98% ee with **2a**′ and 94% ee with **2e**′, yielding epoxide **4b**. Despite the *tert*-butyl substitution in substrate **3c** leading to moderate conversion compared to other substrates, the enantioselectivity of the reaction with **2a**′ (99% ee) remained higher than that with **2e**′ (93% ee). Introducing a 4-chloro group on the β-phenyl group of the chalcone reduced the ee values of product **4d** (88% ee with **2a**′ and 65% ee with **2e**′). Conversely, incorporating a 4-methoxy group yielded epoxide **4e** with exceptional enantioselectivities of 99% ee with **2a**′ and 97% ee with **2e**′. Overall, the α-carbocyclic dAAs-containing peptide **2a**′ demonstrated higher yields and enantioselectivities across all substrates **3a**–**3e** compared to the acyclic dAAs-containing peptide **2e**′.

**Figure 2:**
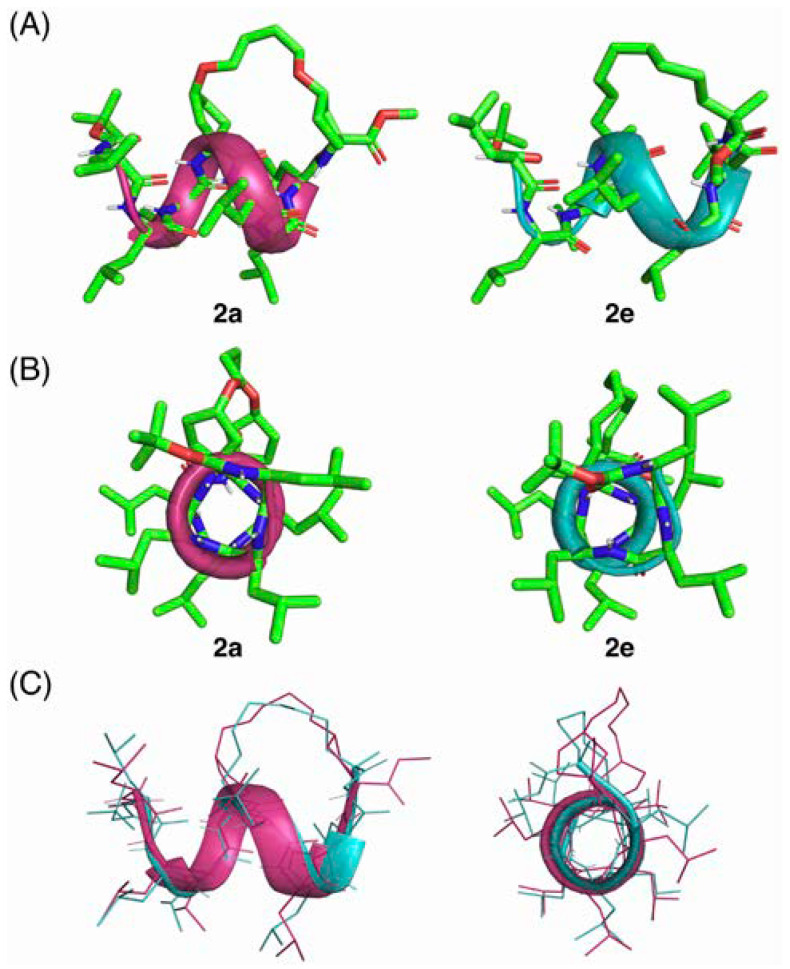
X-ray crystallographic structures of peptides **2a** and **2e** (Mol A). (**A**) A view perpendicular to the helical axis, (**B**) a view along the helical axis, and (**C**) superimposed structures of **2a** (magenta) and **2e** (cyan).

Given the significant influence of peptide catalysts’ secondary structure on the stereochemical outcomes of epoxidation reactions, a thorough conformational investigation of the peptides was conducted. Specifically, X-ray crystallographic analysis was employed to examine peptides **2a** and **2e** following recrystallization through slow evaporation of solvents (MeOH/H_2_O for **2a** and MeOH/EtOAc/*n*-hexane for **2e**) at room temperature. Figure 2 provides an overview of the X-ray crystallographic structures of peptides **2a** and **2e**. The crystal structure of peptide **2a** was resolved in the space group of *P*2_1_, while that of peptide **2e** was resolved in the space group of *P*2_1_2_1_2_1_, containing three crystallographically independent molecules labeled A, B, and C in the asymmetric unit (Figures S1–S3, Tables S1–S6) [48]. Peptide **2a** demonstrated the presence of four consecutive intramolecular hydrogen bonds [N(*n* + 4)H···O=C(*n*)] of the *i* ← *i* + 4 type, indicating the existence of an α-helical structure (Table S2), while peptide **2e** (Mol A) exhibited two sets of consecutive intramolecular hydrogen bonds [N(*n* + 4)H···O=C(*n*)] of the *i* ← *i* + 4 type at the N-terminus and the *i* ← *i* + 3 type at the C-terminus. This suggests a preference for an α-helical structure at the N-terminus and a 3_10_-helical structure at the C-terminus. Additionally, the torsion angles provided insights into the secondary structure of the peptides. The ideal values of the torsion angles for the right-handed α-helix are ϕ = −63° and ψ = −42°, while for the right-handed 3_10_-helix, they are typically ϕ = −57° and ψ = −30° [48,49]. The average torsion angles observed in peptide **2e** were calculated as follows: avg. (ϕ1–ϕ7) = −74.3° (mol A), −72.9° (mol B), and −73.5° (mol C) and avg. (ψ1–ψ7) = −31.8° (mol A), −27.8° (mol B), and −32.3° (mol C), indicating a preference for the right-handed 3_10_-helical structure in peptide **2e**. Conversely, peptide **2a** demonstrated a higher preference for the right-handed α-helical structure, with averaged torsion angles of avg. (ϕ1–ϕ7) = −68.2° and avg. (ψ1–ψ7) = −35.9. In the epoxidation reactions detailed in Figure 1, the α-carbocyclic dAAs-containing peptide **2a**′ exhibited higher enantioselectivities compared to the acyclic dAAs-containing peptide **2e**′. This difference in enantioselectivities could be attributed to their respective preferred peptide secondary structures. In particular, the α-helical structure of the α-carbocyclic dAAs-containing peptide **2a**′ is more effective in asymmetric induction compared to the 3_10_-helical structure of the acyclic dAAs-containing peptide **2e**′ [6,9,11].

**Figure 3:**
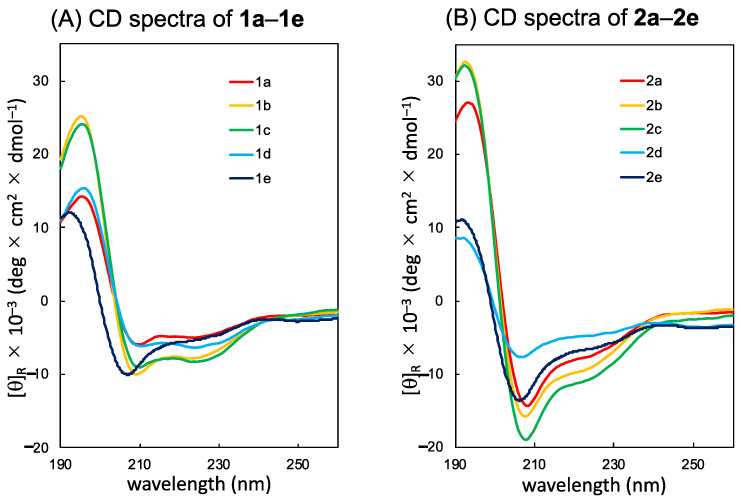
CD spectra of (**A**) unstapled peptides **1a**–**1e** and (**B**) stapled peptides **2a**–**2e**. Conditions: 0.050 mM concentration in 10% MeOH in H_2_O.

The circular dichroism (CD) spectra of unstapled peptides **1a**–**1e** and stapled peptides **2a**–**2e** were analyzed in an aqueous solution containing 10% MeOH (Figure 3 and Figure S4). The CD spectra indicated that all stapled peptides **2a**–**2e** displayed an increased right-handed helicity, accompanied by the presence of α- and 3_10_-helices, in contrast to the corresponding unstapled peptides **1a**–**1e**. Consequently, the introduction of stapled helical peptide organocatalysts could offer advantages for reactions in aqueous media by stabilizing the helical secondary structures of the peptides.

**Figure 4:**
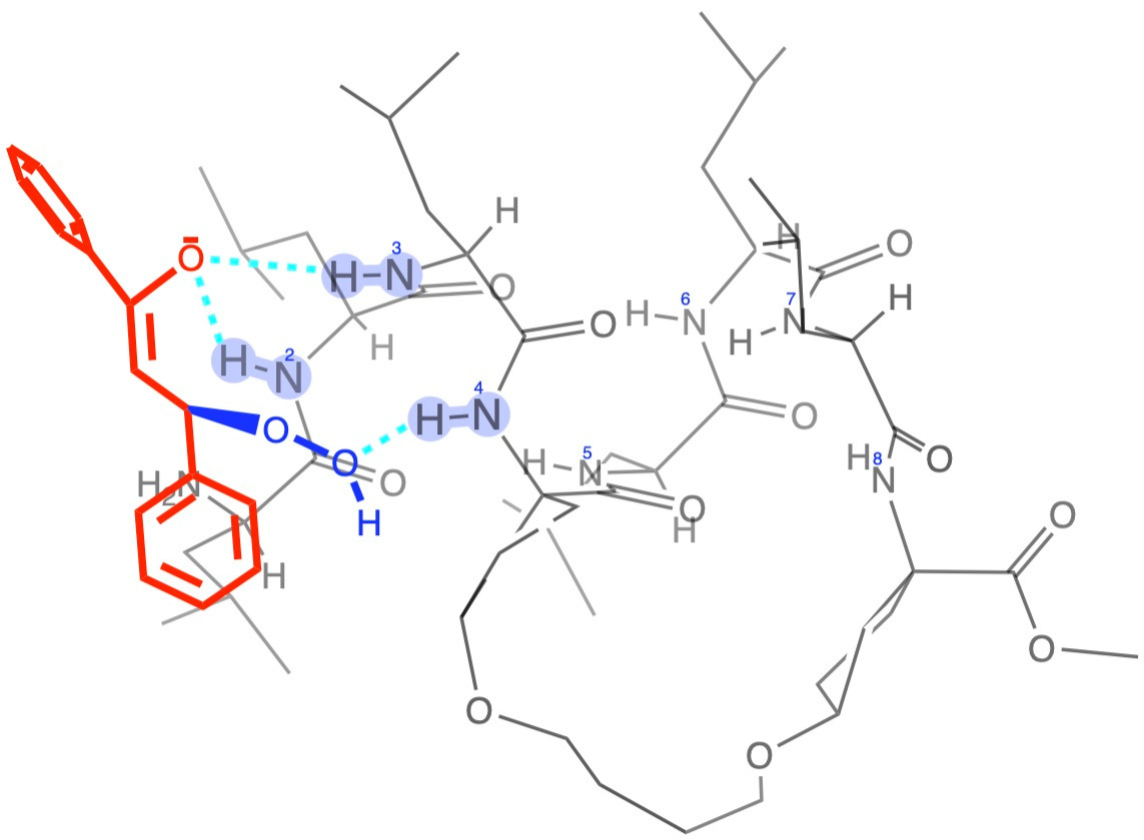
Plausible mechanism for the asymmetric epoxidation of **3a** catalyzed by peptide **2a**′.

Based on the Roberts model [50] and X-ray crystallographic analysis of peptide **2a**, a plausible mechanism for the asymmetric epoxidation of chalcone **3a** catalyzed by peptide catalyst **2a**′ is illustrated in Figure 4. Initially, a reversible nucleophilic addition of hydroperoxide to chalcone **3a** leads to the formation of (*R*)-configured peroxyenolate. The peroxyenolate can then bind to free amidic protons at N(2)H and N(3)H with enolate oxygen (red) and at N(4)H with hydroperoxide oxygen (blue). Finally, the enolate attacks the hydroperoxide moiety, releasing (2*R*,3*S*)-epoxide **4a**.

## 3. Materials and Methods

### 3.1. General Procedure and Method

**Figure 5:**
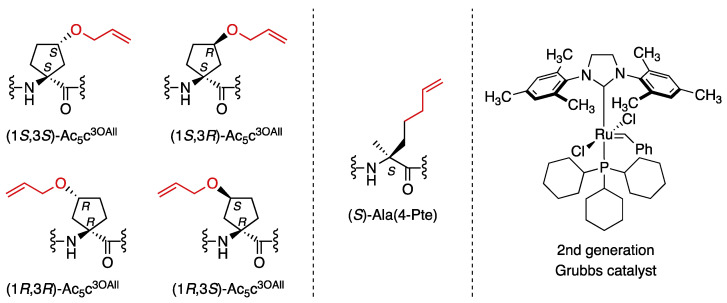
Structures of the second-generation Grubbs catalyst and olefin-tethered α-carbocyclic and acyclic dAAs used in this study.

The melting points were measured using an AS ONE melting point apparatus ATM-01 (AS ONE Corporation, Osaka, Japan) and were uncorrected. Optical rotations were measured using CHCl_3_ or MeOH as solvents on a JASCO DIP-370 polarimeter (JASCO Corporation, Tokyo, Japan). ^1^H NMR and ^13^C{^1^H} NMR spectra were recorded on JEOL JNM-AL-400 (400 MHz and 100 MHz; JEOL Ltd., Tokyo, Japan), Varian NMR System 500PS SN (500 MHz and 125 MHz; Agilent Inc., Santa Clara, USA), or Varian Gemini 300 (300 MHz; Agilent Inc., Santa Clara, USA) spectrometers. Chemical shifts (δ) are reported in parts per million (ppm). For ^1^H NMR spectra (CDCl_3_), tetramethylsilane was used as the internal reference (0.00 ppm), while the central solvent peak was used as the reference (77.0 ppm in CDCl_3_) for ^13^C{^1^H} NMR spectra. Infrared (IR) spectra were acquired using a Shimadzu IRAffinity-1 FT-IR spectrophotometer (Shimadzu Corporation, Kyoto, Japan). High-resolution mass spectra (HRMS) were obtained on a JEOL JMS-T100TD (JEOL Ltd., Tokyo, Japan) using electrospray ionization (ESI) or direct analysis in real-time (DART) ionization in time-of-flight (TOF) mode. Circular dichroism (CD) spectra were measured with a JASCO J-725N spectropolarimeter (JASCO Corporation, Tokyo, Japan) using a 1.0 mm path length cell. Analytical and semi-preparative thin layer chromatography (TLC) was performed with Merck Millipore pre-coated TLC plates (MilliporeSigma, Burlington, United States), silica gel 60 F_254_, with layer thicknesses of 0.25 and 0.50 mm, respectively. The compounds were observed under UV light at 254 nm and then visualized by staining with iodine, *p*-anisaldehyde, or phosphomolybdic acid. Flash and gravity column chromatography separations were performed on Kanto Chemical (Kanto Chemical Co.,Inc., Tokyo, Japan) silica gel 60N and spherical neutral particles with sizes of 40–50 μm and 63–210 μm, respectively. Analytical high-performance liquid chromatography (HPLC) was carried out with a UV spectrophotometric detector (254 nm), to which a 4.6 × 250 mm size chiral column (Daicel Chiralpak AD-H or IB N-5, Daicel Corporation, Osaka, Japan) was attached. All moisture-sensitive reactions were carried out in an inert atmosphere. The reagents and solvents were of commercial grade and were used as supplied, unless otherwise noted. Structures of the second-generation Grubbs catalyst and olefin-tethered α-carbocyclic and acyclic dAAs are shown in Figure 5. Peptides **1a**–**1e** were prepared by liquid-phase peptide synthesis according to a previous report [45]. The α,β-unsaturated ketones **4a**–**4e** were prepared according to a previous report [14].

### 3.2. General Procedure for Peptide-Catalyzed Asymmetric Epoxidation

A mixture of the peptide catalyst (5.23 mg for **1a**′–**1d**′, 4.95 mg for **1e**′, 5.10 mg for **2a**′–**2d**′, 4.82 mg for **2e**′, 5.00 μmol) and chalcone (0.100 mmol) was dissolved in THF (0.50 mL). Urea–hydrogen peroxide (10.3 mg, 0.110 mmol) and 1,8-diazabicyclo[5.4.0]undec-7-ene (DBU; 0.0836 mL, 0.560 mmol) were added to the mixture at 0 °C, and the mixture was gradually warmed to room temperature. After stirring for 24 h, the reaction mixture was diluted with EtOAc and washed with 10% aqueous Na_2_S_2_O_3_. The organic layer was concentrated under vacuum to obtain a residue, which was purified by flash column chromatography on silica gel to yield the desired epoxide.

### 3.3. Synthesis of Stapled Peptides ***2a***–***2e***

*Boc-(L-Leu)_3_-(1S,3S)-Ac_5_c^3OT^-(L-Leu)_3_-(1R,3S)-Ac_5_c^3OT^′-OMe* (**2a**, T,T′ = butyl tether): to a solution of octapeptide **1a** (102 mg, 89.1 μmol) in CH_2_Cl_2_ (17 mL), the second-generation Grubbs catalyst (14.9 mg, 17.5 μmol) was added at room temperature. The resultant mixture was stirred at room temperature for 4 h before being passed through a short plug of silica gel and eluted with EtOAc. Evaporation of the solvent gave a residue, which was used for the next step without further purification. The crude product was dissolved in THF (10 mL), and 5% palladium on carbon (25 mg) was added under a nitrogen atmosphere. The reaction mixture was vigorously stirred overnight at room temperature under a hydrogen atmosphere. The resulting black suspension was filtered through a Celite pad and washed with THF. After removing the solvent under vacuum, the residue was purified by flash column chromatography on silica gel (90% EtOAc in *n*-hexane) to yield the desired product **2a** (92.4 mg, 93% in two steps) as a white amorphous solid. Mp: 248–250 °C. [α]^22^_D_: +6.3 (*c* 1.00, CHCl_3_). ^1^H NMR (500 MHz, CDCl_3_) δ: 7.67 (d, *J* = 8.4 Hz, 1H), 7.58 (br s, 1H), 7.53 (br s, 1H), 7.50 (d, *J* = 5.8 Hz, 1H), 7.27 (d, *J* = 6.1 Hz, 1H), 7.25 (d, *J* = 4.5 Hz, 1H), 7.17 (d, *J* = 2.5 Hz, 1H), 6.07 (br s, 1H), 4.46 (ddd, *J* = 12.0, 8.4, 3.5 Hz, 1H), 4.24 (ddd, *J* = 11.0, 6.0, 3.3 Hz, 1H), 4.17–4.02 (m, 3H), 4.02–3.95 (m, 1H), 3.95–3.86 (m, 2H), 3.68 (s, 3H), 3.44–3.32 (m, 1H), 3.32–3.23 (m, 1H), 3.22–3.04 (m, 2H), 2.70 (s, 1H), 2.63 (dd, *J* = 14.5, 6.0 Hz, 1H), 2.56 (dd, *J* = 13.5, 6.1 Hz, 1H), 2.32–2.24 (m, 2H), 2.08–1.56 (m, 26H), 1.49 (s, 9H), 1.47–1.33 (m, 3H), 1.02–0.85 (m, 36H). ^13^C{^1^H} NMR (125 MHz, CDCl_3_) δ: 175.2, 175.1, 174.93, 174.90, 174.1, 173.7, 173.5, 173.3, 157.3, 81.1, 79.9, 78.6, 68.3, 67.3, 65.2, 64.9, 55.5, 54.9, 54.6, 54.4, 54.1, 52.3, 51.6, 43.7, 40.6, 40.1, 40.0, 39.8, 39.7, 39.4 (2C), 35.5, 33.2, 32.4, 31.9, 28.2 (3C), 27.5, 26.2, 25.1, 24.9, 24.8 (2C), 24.7, 24.5, 23.5, 23.4, 23.3, 22.8, 22.7, 22.5, 21.8 (2C), 21.2, 21.0 (2C), 20.8. IR (KBr): 3329, 2959, 1655, 1535 cm^–1^. HRMS (ESI) *m*/*z*: [M + Na]^+^ calcd for C_58_H_102_N_8_O_13_Na, 1141.7459; found, 1141.7466. CCDC 2341454 contains the supplementary crystallographic data for this paper. These data can be obtained free of charge via https://www.ccdc.cam.ac.uk/ (or from the CCDC, 12 Union Road, Cambridge CB2 1EZ, UK; Fax: +44 1223 336033; E-mail: deposit@ccdc.cam.ac.uk).

*Boc-(L-Leu)_3_-(1R,3R)-Ac_5_c^3OT^-(L-Leu)_3_-(1R,3S)-Ac_5_c^3OT^′-OMe* (**2b**, T,T′ = butyl tether): compound **2b** (76.7 mg) was synthesized from octapeptide **1b** (101 mg, 88.2 μmol) in 75% yield in two steps under the same conditions as described for the synthesis of compound **2a**. The starting material **1b** (24.2 mg, 24%rsm) was recovered after the first step. Eluent for column: 90% EtOAc in *n*-hexane. White solid. Mp: 250–252 °C. [α]^24^_D_: +21.5 (*c* 1.00, CHCl_3_). ^1^H NMR (500 MHz, CDCl_3_) δ: 7.59 (br s, 1H), 7.46 (br s, 1H), 7.35 (d, *J* = 8.7 Hz, 1H), 7.33–7.27 (m, 2H), 7.25 (d, *J* = 5.1 Hz, 1H), 7.23 (d, *J* = 6.0 Hz, 1H), 6.23 (br s, 1H), 4.58–4.47 (m, 1H), 4.29 (ddd, *J* = 10.8, 6.4, 4.3 Hz, 1H), 4.15–3.94 (m, 5H), 3.90 (td, *J* = 7.7, 2.9 Hz, 1H), 3.68 (s, 3H), 3.51–3.43 (m, 1H), 3.43–3.25 (m, 3H), 3.17 (d, *J* = 15.0 Hz, 1H), 2.92 (dd, *J* = 14.4, 6.9 Hz, 1H), 2.66 (br s, 1H), 2.48 (dt, *J* = 13.3, 7.6 Hz, 1H), 2.41–2.30 (m, 1H), 2.13 (dd, *J* = 14.5, 3.6 Hz, 1H), 2.10–2.01 (m, 2H), 1.99–1.56 (m, 26H), 1.49 (s, 9H), 1.03–0.83 (m, 36H). ^13^C{^1^H} NMR (125 MHz, CDCl_3_) δ: 175.2, 175.1, 174.8, 174.1 (2C), 174.0, 173.6, 173.5, 157.5, 81.0, 79.6, 79.4, 66.8, 66.5, 66.0, 64.7, 55.6, 55.2, 54.9, 54.7, 53.7, 52.4, 52.1, 41.2, 41.1, 40.5, 40.14, 40.11, 40.0, 39.7, 39.6, 35.9, 35.4, 32.8, 30.5, 28.3 (3C), 27.0, 26.7, 24.91, 24.85, 24.78, 24.72, 24.6, 24.5, 23.3, 23.2, 23.1, 22.5, 22.31, 22.27, 22.25, 22.0, 21.5, 21.3, 21.2, 21.0. IR (KBr): 3341, 2959, 1655, 1535 cm^–1^. HRMS (ESI) *m*/*z*: [M + Na]^+^ calcd for C_58_H_102_N_8_O_13_Na, 1141.7459; found, 1141.7466.

*Boc-(L-Leu)_3_-(1S,3S)-Ac_5_c^3OT^-(L-Leu)_3_-(1S,3R)-Ac_5_c^3OT^′-OMe* (**2c**, T,T′ = butyl tether): compound **2c** (48.4 mg) was synthesized from octapeptide **1c** (102 mg, 89.0 μmol) in 49% yield in two steps under the same conditions as described for the synthesis of compound **2a**. The starting material **1c** (42.7 mg, 42%rsm) was recovered after the first step. Eluent for column: 90% EtOAc in *n*-hexane. White solid. Mp: 133–135 °C. [α]^23^_D_: +0.4 (*c* 1.00, CHCl_3_). ^1^H NMR (500 MHz, CDCl_3_) δ: 7.57 (br s, 1H), 7.51 (d, *J* = 8.2 Hz, 1H), 7.46 (br s, 1H), 7.40 (d, *J* = 6.9 Hz, 1H), 7.23 (d, *J* = 5.9 Hz, 1H), 7.18 (d, *J* = 4.8 Hz, 1H), 7.09 (d, *J* = 3.9 Hz, 1H), 5.96 (d, *J* = 2.9 Hz, 1H), 4.40 (ddd, *J* = 10.6, 8.3, 5.0 Hz, 1H), 4.28 (ddd, *J* = 11.0, 6.7, 3.6 Hz, 1H), 4.17–4.09 (m, 1H), 4.08–4.02 (m, 2H), 3.97–3.87 (m, 3H), 3.70 (s, 3H), 3.39–3.32 (m, 2H), 3.26–3.20 (m, 1H), 3.16 (td, *J* = 8.5, 3.8 Hz, 1H), 3.03 (dt, *J* = 13.0, 8.5 Hz, 1H), 2.75 (ddd, *J* = 12.6, 8.1, 3.8 Hz, 1H), 2.53 (d, *J* = 14.5 Hz, 1H), 2.35 (dd, *J* = 14.7, 5.4 Hz, 1H), 2.33–2.22 (m, 2H), 2.13–2.03 (m, 2H), 2.01–1.92 (m, 2H), 1.86–1.73 (m, 13H), 1.70–1.60 (m, 11H), 1.49 (s, 9H), 0.99–0.88 (m, 36H). ^13^C{^1^H} NMR (125 MHz, CDCl_3_) δ: 175.7, 175.0, 174.9, 174.1, 174.0, 173.7, 173.5, 173.3, 157.3, 81.1, 79.8, 78.2, 67.8, 66.9, 65.5, 64.4, 55.5, 54.72, 54.69, 54.4, 53.7, 53.0, 52.4, 44.32, 44.26, 40.8, 40.10, 40.07, 39.7, 39.61, 39.56, 35.5, 32.9, 31.7 (2C), 28.2 (3C), 27.7, 26.5, 25.2, 24.95, 24.86, 24.77, 24.71, 24.6, 23.5, 23.3, 23.1, 22.8, 22.6, 22.5, 22.0, 21.9, 21.5, 21.3, 21.1, 20.9. IR (KBr): 3337, 2959, 1659, 1535 cm^–1^. HRMS (ESI) *m*/*z*: [M + Na]^+^ calcd for C_58_H_102_N_8_O_13_Na, 1141.7459; found, 1141.7468.

*Boc-(L-Leu)_3_-(1R,3R)-Ac_5_c^3OT^-(L-Leu)_3_-(1S,3R)-Ac_5_c^3OT^′-OMe* (**2d**, T,T′ = butyl tether): compound **2d** (35.3 mg) was synthesized from octapeptide **1d** (116 mg, 101 μmol) in 30% yield in two steps under the same conditions as described for the synthesis of compound **2a**. The starting material **1d** (67.8 mg, 58%rsm) was recovered after the first step. Eluent for column: 85% EtOAc in *n*-hexane. White solid. Mp: 252–254 °C dec. [α]^22^_D_: +12.4 (*c* 1.00, CHCl_3_). ^1^H NMR (500 MHz, CDCl_3_) δ: 7.78 (br s, 1H), 7.51 (br s, 1H), 7.36 (d, *J* = 6.1 Hz, 1H), 7.33 (d, *J* = 8.7 Hz, 1H), 7.31–7.27 (m, 2H), 7.23 (d, *J* = 8.0 Hz, 1H), 6.17 (br s, 1H), 4.47–4.40 (m, 2H), 4.04–3.94 (m, 4H), 3.92–3.84 (m, 2H), 3.75 (s, 3H), 3.63–3.51 (m, 2H), 3.37–3.29 (m, 2H), 3.27–3.21 (m, 1H), 3.08 (d, *J* = 14.9 Hz, 1H), 2.73–2.66 (m, 1H), 2.35 (d, *J* = 14.5 Hz, 2H), 2.20 (br s, 1H), 2.16–2.10 (m, 2H), 2.05–1.97 (m, 3H), 1.92–1.87 (m, 4H), 1.81–1.72 (m, 12H), 1.66–1.61 (m, 7H), 1.49 (s, 9H), 0.96–0.88 (m, 36H). ^13^C{^1^H} NMR (125 MHz, CDCl_3_) δ: 175.7, 175.5, 175.1, 174.4, 174.0, 173.0, 172.7, 172.6, 157.4, 81.0, 80.2, 79.7, 67.7, 67.6, 66.4, 64.6, 55.6, 54.9, 54.6, 54.4, 53.5, 52.8, 52.6, 43.2, 41.6, 41.2, 40.2, 40.1 (2C), 39.6, 39.2, 36.0, 35.9, 33.2, 32.8, 28.3 (3C), 27.3, 26.5, 25.0, 24.9, 24.81, 24.76, 24.74, 24.6, 23.4, 23.3, 23.0, 22.7, 22.5, 22.4, 22.2, 22.0, 21.8, 21.4, 21.2, 20.8. IR (KBr): 3341, 2959, 1655, 1535 cm^–1^. HRMS (ESI) *m*/*z*: [M + Na]^+^ calcd for C_58_H_102_N_8_O_13_Na, 1141.7459; found, 1141.7468.

*Boc-(L-Leu)_3_-(S)-Ala(4-Bte^T^)-(L-Leu)_3_-(S)-Ala(4-Bte^T^′)-OMe* (**2e**: T,T′ = tether): to a solution of octapeptide **1a** (147 mg, 0.135 mmol) in CH_2_Cl_2_ (27 mL), the second-generation Grubbs catalyst (22.9 mg, 27.0 μmol) was added at room temperature, and the resultant solution was stirred at room temperature for 24 h. The reaction mixture was passed through a short plug of silica gel, eluted with CHCl_3_ (discarded), followed by EtOAc (collected), and concentrated. The ^1^H NMR spectrum of the crude product revealed 49% conversion with a 44:56 ratio of *E* and *Z* isomers [*E* isomer: *R*_f_ = 0.43 (80% EtOAc in *n*-hexane); *Z* isomer: *R*_f_ = 0.30 (80% EtOAc in *n*-hexane)]. The residue was purified by flash column chromatography on silica gel (60–80% EtOAc in *n*-hexane) to produce crude product **S-1**, which was used for the next step without further purification. A suspension of the above octapeptide **S-1** and 10% Pd/C (36 mg) in MeOH (1 mL) was stirred at room temperature for 2 d under a hydrogen atmosphere. After filtration through a Celite pad and concentration under vacuum, the obtained residue was purified by flash column chromatography on silica gel (70% EtOAc in *n*-hexane) to yield **2e** (62.4 mg, 43% in two steps) as a white solid. *R*_f_ = 0.37 (80% EtOAc in *n*-hexane). Mp: 241–243 °C. [α]^25^_D_: –12.0 (*c* 1.00, CHCl_3_). ^1^H NMR (500 MHz, CDCl_3_) δ: 7.49 (d, *J* = 7.2 Hz, 1H), 7.45 (d, *J* = 4.8 Hz, 1H), 7.42 (d, *J* = 9.4 Hz, 1H), 7.38 (br s, 1H), 7.29 (d, *J* = 5.4 Hz, 1H), 7.22 (br s, 1H), 6.91 (d, *J* = 4.2 Hz, 1H), 5.77 (d, *J* = 2.8 Hz, 1H), 4.55 (td, *J* = 9.3, 6.0 Hz, 1H), 4.34 (ddd, *J* = 11.3, 7.2, 3.6 Hz, 1H), 4.09–4.03 (m, 1H), 4.03–3.96 (m, 2H), 3.94 (ddd, *J* = 8.9, 6.1, 2.8 Hz, 1H), 3.73 (s, 3H), 1.95–1.54 (m, 20H), 1.61 (s, 6H), 1.49 (s, 9H), 1.49–1.16 (m, 14H), 1.02–0.93 (m, 21H), 0.93–0.84 (m, 15H). ^13^C{^1^H} NMR (125 MHz, CDCl_3_) δ: 176.4, 175.2, 174.3, 173.6, 173.42, 173.39, 173.34, 172.7, 157.2, 81.3, 60.0, 59.6, 55.3, 54.8, 54.7, 54.4, 53.4, 52.9, 52.4, 41.4, 40.2, 40.1, 39.79, 39.76, 39.6, 38.8, 38.7, 28.2 (3C), 26.8, 26.6, 26.5, 26.4, 25.00, 24.97, 24.95, 24.89, 24.7, 24.6, 23.55, 23.50, 23.0, 22.88, 22.85, 22.77, 22.5, 22.4, 21.84, 21.77, 21.5, 21.4, 21.2, 21.1, 20.8, 20.6. IR (KBr): 3325, 2957, 1755, 1647 cm^–1^. HRMS (ESI) *m*/*z*: [M + Na]^+^ calcd for C_56_H_102_N_8_O_11_Na, 1085.7566; found, 1085.7547. CCDC 2341476 contains the supplementary crystallographic data for this paper. These data can be obtained free of charge via https://www.ccdc.cam.ac.uk/ (or from the CCDC, 12 Union Road, Cambridge CB2 1EZ, UK; Fax: +44 1223 336033; E-mail: deposit@ccdc.cam.ac.uk).

### 3.4. Synthesis of Unstapled Peptide Catalysts ***1a**′*–***1e**′* and Stapled Peptide Catalysts ***2a**′*–***2e**′*

*H-(L-Leu)_3_-(1S,3S)-Ac_5_c^3OAll^-(L-Leu)_3_-(1R,3S)-Ac_5_c^3OAll^-OMe* (**1a**′): to a solution of octapeptide **1a** (20.0 mg, 17.5 μmol) in CH_2_Cl_2_ (0.175 mL), trifluoroacetic acid (17.5 μL) was added at room temperature and stirred at the same temperature for 19 h. Additional trifluoroacetic acid (17.5 μL) was added to the reaction mixture, which was then stirred for 7 h. The reaction was quenched by adding sat. NaHCO_3_ aq (1.5 mL), and the mixture was extracted with EtOAc (2 mL × 5). The combined organic layers were dried over anhydrous Na_2_SO_4_ and concentrated under vacuum. The residue was purified by column chromatography on silica gel (5% MeOH in CHCl_3_) to give the title compound **1a**′ (17.5 mg) in 96% yield as a white solid. ^1^H NMR (500 MHz, CDCl_3_) δ: 8.23 (br s, 1H), 7.73 (s, 1H), 7.55 (s, 1H), 7.48 (d, *J* = 6.4 Hz, 1H), 7.39 (d, *J* = 5.1 Hz, 1H), 7.30 (d, *J* = 8.4 Hz, 1H), 6.81 (d, *J* = 3.5 Hz, 1H), 5.93–5.83 (m, 2H), 5.26 (dq, *J* = 15.1, 1.7 Hz, 1H), 5.23 (dq, *J* = 15.1, 1.7 Hz, 1H), 5.14 (dq, *J* = 10.4, 1.3 Hz, 1H), 5.10 (dq, *J* = 10.4, 1.3 Hz, 1H), 4.48–4.40 (m, 1H), 4.25–4.20 (m, 1H), 4.19–4.14 (m, 1H), 4.12–4.03 (m, 2H), 4.03–3.92 (m, 5H), 3.88 (ddt, *J* = 12.8, 5.7, 1.5 Hz, 1H), 3.67 (s, 3H), 3.48 (dd, *J* = 9.7, 4.1 Hz, 1H), 2.85–2.75 (m, 2H), 2.63 (dd, *J* = 13.8, 6.4 Hz, 1H), 2.40–2.33 (m, 1H), 2.16–2.09 (m, 1H), 2.02 (dd, *J* = 14.1, 5.3 Hz, 1H), 1.99–1.85 (m, 7H), 1.85–1.73 (m, 10H), 1.73–1.63 (m, 6H), 1.61 (dd, *J* = 8.3, 5.4 Hz, 1H), 1.57–1.49 (m, 1H), 1.40 (ddd, *J* = 14.2, 9.7, 4.6 Hz, 1H), 1.01 (d, *J* = 6.2 Hz, 3H), 0.99–0.93 (m, 18H), 0.92–0.89 (m, 9H), 0.88 (d, *J* = 6.7 Hz, 3H), 0.86 (d, *J* = 6.1 Hz, 3H). HRMS (ESI) *m*/*z*: [M + Na]^+^ calcd for C_55_H_96_N_8_O_11_Na, 1067.7091; found, 1067.7089.

*H-(L-Leu)_3_-(1R,3R)-Ac_5_c^3OAll^-(L-Leu)_3_-(1R,3S)-Ac_5_c^3OAll^-OMe* (**1b**′): compound **1b**′ (18.2 mg) was synthesized from octapeptide **1b** (20.0 mg, 17.5 μmol) in quantitative yield under the same conditions as described for the synthesis of compound **1a**′. Eluent for column: 5% MeOH in CHCl_3_. White solid. ^1^H NMR (500 MHz, CDCl_3_) δ: 8.22 (br s, 1H), 7.65 (s, 1H), 7.56 (s, 1H), 7.49 (d, *J* = 6.1 Hz, 1H), 7.37 (d, *J* = 5.3 Hz, 1H), 7.30 (d, *J* = 8.4 Hz, 1H), 6.87 (d, *J* = 2.8 Hz, 1H), 5.92–5.81 (m, 2H), 5.27–5.19 (m, 2H), 5.15–5.07 (m, 2H), 4.47–4.39 (m, 1H), 4.25–4.18 (m, 1H), 4.12–4.03 (m, 2H), 4.00–3.91 (m, 5H), 3.91–3.85 (m, 2H), 3.67 (s, 3H), 3.46 (dd, *J* = 8.9, 4.4 Hz, 1H), 2.80 (dd, *J* = 14.1, 6.7 Hz, 1H), 2.64 (dd, *J* = 13.9, 7.1 Hz, 1H), 2.41–2.29 (m, 2H), 2.20–2.04 (m, 4H), 2.04–1.93 (m, 4H), 1.92–1.66 (m, 18H), 1.62–1.51 (m, 1H), 1.38 (ddd, *J* = 14.1, 9.4, 5.0 Hz, 1H), 1.01 (d, *J* = 6.4 Hz, 3H), 0.99–0.93 (m, 21H), 0.92–0.86 (m, 12H). HRMS (ESI) *m*/*z*: [M + Na]^+^ calcd for C_55_H_96_N_8_O_11_Na, 1067.7091; found, 1067.7097.

*H-(L-Leu)_3_-(1S,3S)-Ac_5_c^3OAll^-(L-Leu)_3_-(1S,3R)-Ac_5_c^3OAll^-OMe* (**1c**′): compound **1c**′ (18.2 mg) was synthesized from octapeptide **1c** (20.0 mg, 17.5 μmol) in quantitative yield under the same conditions as described for the synthesis of compound **1a**′. Eluent for column: 5% MeOH in CHCl_3_. White solid. ^1^H NMR (500 MHz, CDCl_3_) δ: 8.28 (br s, 1H), 7.76 (s, 1H), 7.66 (s, 1H), 7.50 (d, *J* = 6.4 Hz, 1H), 7.45 (d, *J* = 5.3 Hz, 1H), 7.34 (d, *J* = 6.8 Hz, 1H), 7.13 (br s, 1H), 5.94–5.84 (m, 2H), 5.29–5.21 (m, 2H), 5.17–5.10 (m, 2H), 4.45–4.36 (m, 1H), 4.25–4.14 (m, 2H), 4.11–4.04 (m, 2H), 4.01–3.93 (m, 6H), 3.66 (s, 3H), 3.49 (dd, *J* = 9.4, 4.5 Hz, 1H), 2.87–2.78 (m, 2H), 2.65 (dd, *J* = 13.8, 6.7 Hz, 1H), 2.32–2.23 (m, 2H), 2.21–2.12 (m, 3H), 2.03 (dd, *J* = 14.1, 6.0 Hz, 1H), 1.98–1.86 (m, 4H), 1.85–1.75 (m, 8H), 1.74–1.66 (m, 8H), 1.61–1.55 (m, 2H), 1.46–1.37 (m, 1H), 1.00 (d, *J* = 6.4 Hz, 3H), 0.98–0.92 (m, 18H), 0.91–0.83 (m, 15H). HRMS (ESI) *m*/*z*: [M + Na]^+^ calcd for C_55_H_96_N_8_O_11_Na, 1067.7091; found, 1067.7083.

*H-(L-Leu)_3_-(1R,3R)-Ac_5_c^3OAll^-(L-Leu)_3_-(1S,3R)-Ac_5_c^3OAll^-OMe* (**1d**′): compound **1d**′ (15.8 mg) was synthesized from octapeptide **1d** (20.0 mg, 17.5 μmol) in 86% yield under the same conditions as described for the synthesis of compound **1a**′. Eluent for column: 5% MeOH in CHCl_3_. White solid. ^1^H NMR (500 MHz, CDCl_3_) δ: 8.23 (br s, 1H), 7.67 (s, 1H), 7.62 (s, 1H), 7.47 (d, *J* = 6.1 Hz, 1H), 7.40 (d, *J* = 5.1 Hz, 1H), 7.30 (d, *J* = 8.2 Hz, 1H), 7.07 (br s, 1H), 5.94–5.81 (m, 2H), 5.25 (dq, *J* = 17.2, 1.7 Hz, 1H), 5.23 (dq, *J* = 17.2, 1.7 Hz, 1H), 5.12 (dt, *J* = 10.5, 1.6 Hz, 2H), 4.45–4.37 (m, 1H), 4.25–4.18 (m, 1H), 4.11–4.04 (m, 2H), 4.00–3.92 (m, 5H), 3.88 (ddt, *J* = 12.7, 5.6, 1.5 Hz, 1H), 3.65 (s, 3H), 3.46 (dd, *J* = 9.1, 4.7 Hz, 1H), 2.82 (dd, *J* = 14.1, 7.0 Hz, 1H), 2.69 (dd, *J* = 14.1, 6.6 Hz, 1H), 2.33–2.23 (m, 2H), 2.23–2.13 (m, 3H), 2.14–1.99 (m, 6H), 1.97–1.90 (m, 1H), 1.90–1.67 (m, 16H), 1.62–1.55 (m, 2H), 1.42–1.35 (m, 1H), 1.00 (d, *J* = 6.2 Hz, 3H), 0.98–0.92 (m, 21H), 0.92–0.85 (m, 12H). HRMS (ESI) *m*/*z*: [M + Na]^+^ calcd for C_55_H_96_N_8_O_11_Na, 1067.7091; found, 1067.7090.

*H-(L-Leu)_3_-(S)-Ala(4-Pte)-(L-Leu)_3_-(S)-Ala(4-Pte)-OMe* (**1e**′): compound **1e**′ (18.2 mg) was synthesized from octapeptide **1e** (20.0 mg, 18.4 μmol) in quantitative yield under the same conditions as described for the synthesis of compound **1a**′. Eluent for column: 5% MeOH in CHCl_3_. White solid. ^1^H NMR (500 MHz, CDCl_3_) δ: 8.11 (br s, 1H), 7.65 (d, *J* = 6.5 Hz, 1H), 7.37 (d, *J* = 5.3 Hz, 1H), 7.31 (br s, 1H), 7.24 (d, *J* = 8.3 Hz, 1H), 7.19 (br s, 1H), 7.00 (br s, 1H), 5.87–5.69 (m, 2H), 5.02–4.89 (m, 4H), 4.48–4.39 (m, 1H), 4.26–4.18 (m, 1H), 4.08–3.98 (m, 2H), 3.97–3.90 (m, 1H), 3.69 (s, 3H), 3.51 (dd, *J* = 9.5, 4.2 Hz, 1H), 2.38–2.12 (m, 2H), 2.09–2.01 (m, 4H), 2.00–1.86 (m, 2H), 1.86–1.78 (m, 4H), 1.77–1.66 (m, 11H), 1.65–1.51 (m, 3H), 1.48 (s, 3H), 1.47 (s, 3H), 1.44–1.36 (m, 4H), 1.03–0.95 (m, 15H), 0.95–0.92 (m, 6H), 0.91–0.85 (m, 15H). HRMS (ESI) *m*/*z*: [M + Na]^+^ calcd for C_53_H_96_N_8_O_9_Na, 1011.7192; found, 1011.7193.

*H-(L-Leu)_3_-(1S,3S)-Ac_5_c^3OT^-(L-Leu)_3_-(1R,3S)-Ac_5_c^3OT^′-OMe* (**2a**′, T,T′ = butyl tether): compound **2a**′ (17.6 mg) was synthesized from octapeptide **2a** (20.0 mg, 17.9 μmol) in 96% yield under the same conditions as described for the synthesis of compound **1a**′. Eluent for column: 8% MeOH in CHCl_3_. White solid. ^1^H NMR (500 MHz, CDCl_3_) δ: 8.34 (br s, 1H), 7.77 (br s, 1H), 7.72–7.47 (m, 3H), 7.46–7.28 (m, 1H), 7.15 (br s, 1H), 4.42–4.27 (m, 1H), 4.24–4.14 (m, 1H), 4.12–3.95 (m, 4H), 3.90 (br s, 1H), 3.70 (s, 3H), 3.62–3.50 (m, 1H), 3.39–3.31 (m, 1H), 3.30–3.24 (m, 1H), 3.20–3.13 (m, 1H), 3.03 (q, *J* = 10.3 Hz, 1H), 2.64 (dd, *J* = 14.5, 5.6 Hz, 1H), 2.54–2.46 (m, 1H), 2.43–2.17 (m, 5H), 2.02–1.91 (m, 3H), 1.89–1.80 (m, 5H), 1.79–1.52 (m, 18H), 1.45 (s, 3H), 1.01–0.97 (m, 6H), 0.97–0.91 (m, 18H), 0.91–0.87 (m, 9H), 0.86–0.84 (m, 3H). HRMS (ESI) *m*/*z*: [M + Na]^+^ calcd for C_53_H_94_N_8_O_11_Na, 1041.6934; found, 1041.6933.

*H-(L-Leu)_3_-(1R,3R)-Ac_5_c^3OT^-(L-Leu)_3_-(1R,3S)-Ac_5_c^3OT^′-OMe* (**2b**′, T,T′ = butyl tether): compound **2b**′ (18.2 mg) was synthesized from octapeptide **2b** (20.0 mg, 17.9 μmol) in quantitative yield under the same conditions as described for the synthesis of compound **1a**′. Eluent for column: 8% MeOH in CHCl_3_. White solid. ^1^H NMR (500 MHz, CDCl_3_) δ: 8.46 (br s, 1H), 8.25–7.75 (m, 5H), 7.66 (br s, 1H), 4.32–4.24 (m, 1H), 4.22–4.14 (m, 1H), 4.08 (s, 1H), 4.00–3.93 (m, 4H), 3.76 (s, 3H), 3.57 (dd, *J* = 8.9, 5.2 Hz, 1H), 3.48–3.38 (m, 2H), 3.35–3.30 (m, 2H), 3.11 (d, *J* = 16.1 Hz, 1H), 2.88 (dd, *J* = 14.6, 7.0 Hz, 1H), 2.46 (dt, *J* = 13.8, 7.8 Hz, 1H), 2.30–2.24 (m, 1H), 2.10–2.01 (m, 3H), 1.94–1.89 (m, 2H), 1.86–1.79 (m, 7H), 1.77–1.72 (m, 5H), 1.70–1.60 (m, 8H), 1.58–1.51 (m, 4H), 1.44–1.30 (m, 3H), 0.99 (d, *J* = 6.1 Hz, 3H), 0.95–0.93 (m, 6H), 0.93–0.91 (m, 9H), 0.90–0.88 (m, 9H), 0.88–0.85 (m, 9H). HRMS (ESI) *m*/*z*: [M + Na]^+^ calcd for C_53_H_94_N_8_O_11_Na, 1041.6934; found, 1041.6936.

*H-(L-Leu)_3_-(1S,3S)-Ac_5_c^3OT^-(L-Leu)_3_-(1S,3R)-Ac_5_c^3OT^′-OMe* (**2c**′, T,T′ = butyl tether): compound **2c**′ (18.2 mg) was synthesized from octapeptide **2c** (20.0 mg, 17.9 μmol) in quantitative yield under the same conditions as described for the synthesis of compound **1a**′. Eluent for column: 8% MeOH in CHCl_3_. White solid. ^1^H NMR (500 MHz, CDCl_3_) δ: 8.44 (1H, br s), 8.16–7.74 (m, 5H), 7.57 (br s, 1H), 4.27–4.19 (m, 1H), 4.19–4.10 (m, 2H), 4.04–3.98 (m, 2H), 3.96–3.91 (m, 2H), 3.77 (s, 3H), 3.70–3.63 (m, 1H), 3.62–3.56 (m, 1H), 3.42–3.33 (m, 2H), 3.28–3.15 (m, 2H), 2.92 (s, 1H), 2.71–2.64 (m, 1H), 2.55 (d, *J* = 13.9 Hz, 1H), 2.26 (s, 1H), 2.15–2.08 (m, 1H), 2.04–1.99 (m, 1H), 1.92–1.83 (m, 6H), 1.80–1.74 (m, 6H), 1.71–1.64 (m, 6H), 1.63–1.56 (m, 6H), 1.53–1.48 (m, 2H), 1.44–1.35 (m, 3H), 0.99 (d, *J* = 6.2 Hz, 3H), 0.96 (d, *J* = 6.6 Hz, 6H), 0.94–0.92 (m, 12H), 0.91–0.90 (m, 3H), 0.89–0.86 (m, 12H). HRMS (ESI) *m*/*z*: [M + Na]^+^ calcd for C_53_H_94_N_8_O_11_Na, 1041.6934; found, 1041.6939.

*H-(L-Leu)_3_-(1R,3R)-Ac_5_c^3OT^-(L-Leu)_3_-(1S,3R)-Ac_5_c^3OT^′-OMe* (**2d**′, T,T′ = butyl tether): compound **2d**′ (17.9 mg) was synthesized from octapeptide **2d** (20.0 mg, 17.9 μmol) in 98% yield under the same conditions as described for the synthesis of compound **1a**′. Eluent for column: 8% MeOH in CHCl_3_. White solid. ^1^H NMR (500 MHz, CDCl_3_) δ: 8.43 (br s, 1H), 8.19–7.74 (m, 5H), 7.59 (br s, 1H), 4.36–4.11 (m, 3H), 4.04–3.89 (m, 6H), 3.80 (s, 3H), 3.69–3.65 (m, 1H), 3.57–3.50 (m, 2H), 3.35–3.26 (m, 2H), 3.26–3.18 (m, 1H), 3.11 (d, *J* = 13.6 Hz, 1H), 2.68–2.61 (m, 1H), 2.55–2.40 (m, 1H), 2.32–2.13 (m, 3H), 2.07–1.99 (m, 3H), 1.95–1.90 (m, 2H), 1.81–1.67 (m, 16H), 1.60–1.52 (m, 6H), 0.98 (d, *J* = 6.1 Hz, 3H), 0.95–0.93 (m, 9H), 0.92–0.90 (m, 12H), 0.89–0.87 (m, 12H). HRMS (ESI) *m*/*z*: [M + Na]^+^ calcd for C_53_H_94_N_8_O_11_Na, 1041.6934; found, 1041.6939.

*H-(L-Leu)_3_-(S)-Ala(4-Bte^T^)-(L-Leu)_3_-(S)-Ala(4-Bte^T^′)-OMe* (**2e**′: T,T′ = tether): compound **2e**′ (18.1 mg) was synthesized from octapeptide **2e** (20.0 mg, 18.8 μmol) in quantitative yield under the same conditions as described for the synthesis of compound **1a**′. Eluent for column: 8% MeOH in CHCl_3_. White solid. ^1^H NMR (500 MHz, CDCl_3_) δ: 8.28 (br s, 1H), 8.07–7.68 (m, 3H), 7.67–7.30 (m, 3H), 4.39 (s, 1H), 4.31–4.21 (m, 1H), 4.06–3.89 (m, 3H), 3.76 (s, 3H), 3.53 (dd, *J* = 9.0, 4.8 Hz, 1H), 2.49–2.15 (m, 3H), 1.99–1.82 (m, 5H), 1.81–1.60 (m, 15H), 1.58 (s, 3H), 1.47 (s, 3H), 1.44–1.17 (m, 13H), 0.99 (d, *J* = 6.2 Hz, 3H), 0.98–0.91 (m, 18H), 0.91–0.85 (m, 15H). HRMS (ESI) *m*/*z*: [M + Na]^+^ calcd for C_51_H_94_N_8_O_9_Na, 985.7036; found, 985.7052.

### 3.5. Synthesis Outlined in Figure 1

*(2R,3S)-trans-Epoxy-3-phenyl-1-phenylpropan-1-one* (**4a**): according to the general procedure for peptide-catalyzed asymmetric epoxidation, the reaction of **3a** (20.8 mg, 0.100 mmol) with catalyst **2a**′ (5.10 mg, 5.00 μmol) yielded **4a** (20.9 mg, 93%) as colorless crystals. Eluent for column: 5% EtOAc in *n*-hexane. *R*_f_ = 0.31 (10% EtOAc in *n*-hexane). [α]^19^_D_ –191.9 (*c* 1.00, CHCl_3_) [Lit., for the antipode, +182.2 (*c* 1.14, CHCl_3_)] [51]. ^1^H NMR (500 MHz, CDCl_3_) δ: 8.15–7.96 (2H, m), 7.70–7.60 (1H, m), 7.57–7.47 (2H, m), 7.47–7.35 (5H, m), 4.31 (1H, d, *J* = 2.0 Hz), 4.08 (1H, d, *J* = 2.0 Hz). HPLC (Chiralpak AD-H, 10% EtOH in *n*-hexane, flow rate = 1.0 mL/min): *t*_R_ = 23.4 min (major), *t*_R_ = 30.0 min (minor), ee = 96%.

*(2R,3S)-trans-Epoxy-3-phenyl-1-(2-furyl)propan-1-one* (**4b**): according to the general procedure for peptide-catalyzed asymmetric epoxidation, the reaction of **3b** (19.8 mg, 0.100 mmol) with catalyst **2a**′ (5.10 mg, 5.00 μmol) yielded **4b** (20.7 mg, 97%) as colorless crystals. Eluent for column: 20% EtOAc in *n*-hexane. *R*_f_ = 0.10 (10% EtOAc in *n*-hexane). [α]^19^_D_ –201.4 (*c* 1.00, CHCl_3_) [Lit., –200 (*c* 1.00, CHCl_3_)] [51]. ^1^H NMR (300 MHz, CDCl_3_) δ: 7.68 (1H, dd, *J* = 1.7, 0.7 Hz), 7.46 (1H, dd, *J* = 3.7, 0.7 Hz), 7.44–7.31 (5H, m), 6.60 (1H, dd, *J* = 3.7, 1.7 Hz), 4.15 (1H, d, *J* = 2.1 Hz), 4.15 (1H, d, *J* = 2.1 Hz). HPLC (Chiralpak IB N-5, 5% EtOH in *n*-hexane, flow rate = 1.0 mL/min): *t*_R_ = 17.5 min (major), *t*_R_ = 16.1 min (minor), ee = 98%.

*(1S,2R)-trans-1,2-Epoxy-4,4-dimethyl-1-phenylpentan-3-one* (**4c**): according to the general procedure for peptide-catalyzed asymmetric epoxidation, the reaction of **3c** (18.8 mg, 0.100 mmol) with catalyst **2a**′ (5.10 mg, 5.00 μmol) yielded **4c** (14.1 mg, 69%) as colorless crystals. Eluent for column: 5% EtOAc in *n*-hexane. *R*_f_ = 0.28 (10% EtOAc in *n*-hexane). [α]^19^_D_ –183.9 (*c* 1.00, CHCl_3_) [Lit., –194 (*c* 1.00, CHCl_3_)] [52]. ^1^H NMR (400 MHz, CDCl_3_) δ: 7.43–7.35 (3H, m), 7.34–7.29 (2H, m), 3.87 (1H, d, *J* = 1.9 Hz), 3.86 (1H, d, *J* = 1.9 Hz), 1.24 (9H, s). HPLC (Chiralpak AD-H, 5% EtOH in *n*-hexane, flow rate = 0.7 mL/min): *t*_R_ = 17.3 min (major), *t*_R_ = 21.4 min (minor), ee = 99%.

*(2R,3S)-trans-Epoxy-3-(4-chlorophenyl)-1-phenylpropan-1-one* (**4d**): according to the general procedure for peptide-catalyzed asymmetric epoxidation, the reaction of **3d** (24.3 mg, 0.100 mmol) with catalyst **2a**′ (5.10 mg, 5.00 μmol) yielded **4d** (24.4 mg, 95%) as yellow crystals. Eluent for column: 5% EtOAc in *n*-hexane. *R*_f_ = 0.35 (10% EtOAc in *n*-hexane). [α]^19^_D_ –176.1 (*c* 1.00, CHCl_3_) [Lit., –233 (*c* 1.00, CH_2_Cl_2_)] [53]. ^1^H NMR (500 MHz, CDCl_3_) δ: 8.08–7.97 (2H, m), 7.69–7.59 (1H, m), 7.56–7.46 (2H, m), 7.44–7.36 (2H, m), 7.35–7.29 (2H, m), 4.26 (1H, d, *J* = 1.8 Hz), 4.06 (1H, d, *J* = 1.8 Hz). HPLC (Chiralpak IB N-5, 2% *i*-propanol in *n*-hexane, flow rate = 1.0 mL/min): *t*_R_ = 25.3 min (major), *t*_R_ = 24.1 min (minor), ee = 88%.

*(2R,3S)-trans-Epoxy-3-(4-methoxyphenyl)-1-phenylpropan-1-one* (**4e**): according to the general procedure for peptide-catalyzed asymmetric epoxidation, the reaction of **3e** (23.8 mg, 0.100 mmol) with catalyst **2a**′ (5.10 mg, 5.00 μmol) yielded **4e** (21.1 mg, 83%) as yellow crystals. Eluent for column: 5% EtOAc in *n*-hexane. *R*_f_ = 0.28 (10% EtOAc in *n*-hexane). [α]^19^_D_ –185.6 (*c* 1.00, CHCl_3_) [Lit., for the antipode, +131 (*c* 0.70, CHCl_3_)] [54]. ^1^H NMR (500 MHz, CDCl_3_) δ: 8.07–7.99 (2H, m), 7.67–7.59 (1H, m), 7.54–7.46 (2H, m), 7.34–7.27 (2H, m), 6.99–6.91 (2H, m), 4.30 (1H, d, *J* = 2.0 Hz), 4.03 (1H, d, *J* = 2.0 Hz), 3.83 (3H, s). HPLC (Chiralpak IB N-5, 2% *i*-propanol in *n*-hexane, flow rate = 1.0 mL/min): *t*_R_ = 33.6 min (major), *t*_R_ = 31.1 min (minor), ee = 99%.

## 4. Conclusions

Peptide foldamer catalysts, featuring a side-chain crosslink, were synthesized via a ring-closing metathesis reaction involving allyloxy groups on the α-carbocyclic dAAs. Stapled and unstapled octapeptide catalysts were prepared and tested via the asymmetric epoxidation of chalcones. However, the enantioselectivities observed in the reactions involving stapled peptides were similar to those of unstapled peptides. Notably, the stapled peptide **2a**′ containing α-carbocyclic dAAs exhibited higher yields and enantioselectivities across all substrates **3a**–**3e** than the acyclic dAAs-containing stapled peptide **2e**′. These results could be attributed to the α-helicities of the peptides. X-ray crystallographic analysis revealed that α-carbocyclic dAAs-containing peptide **2a** favored a right-handed α-helical structure, while acyclic dAAs-containing peptide **2e** formed a mixture of α- and 3_10_-helices. CD spectra of stapled peptides **2a**–**2e** in H_2_O/MeOH (9:1) indicated enhanced right-handed helicities compared to unstapled peptides **1a**–**1e**, potentially advantageous for developing peptide foldamer catalysts in aqueous media. Ongoing research in our laboratory includes further investigations into peptide catalysis in aqueous media and their potential applications in functional peptides.

## Figures and Tables

**Table 1:** Peptide-catalyzed asymmetric epoxidation.

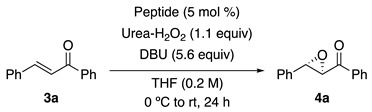			
**Entry**	**Peptide**	**Conv (%) ^1^**	**Ee (%) ^2^**
1	**1a**′	>99	97
2	**2a**′	>99	97
3	**1b**′	>99	98
4	**2b**′	>99	98
5	**1c**′	>99	97
6	**2c**′	>99	98
7	**1d**′	>99	98
8	**2d**′	>99	97
9	**1e**′	>99	89
10	**2e**′	92	90
11	none	71	0

^1^ Conversion was determined by ^1^H NMR analysis. ^2^ Ee was determined by HPLC.

## Data Availability

Data are contained within the article or Supplementary Material.

## Supplementary Materials

The following supporting information can be downloaded at: https://www.mdpi.com/article/10.3390/molecules29184340/s1, Figure S1–S3, Table S1–S6, X-ray crystallographic data of compounds **2a** and **2e**; CD spectra of peptides **1a**–**1e** and **2a**–**2e**; ^1^H and ^13^C NMR spectra of compounds **2a**–**2e**, **1a**′–**1e**′, **2a**′–**2e**′, and **4a**–**4e**; HPLC chart of compounds **4a**–**4e**.
