# Supplementary material for: Conformational Analysis and Organocatalytic Activity of Helical Stapled Peptides Containing α-Carbocyclic α,α-Disubstituted α-Amino Acids
Source: Molecules. 2024 Sep 12;29(18):4340. doi: 10.3390/molecules29184340 (PMC11434043; doi:10.3390/molecules29184340)
Supplement: Supplementary file 1 [file molecules-29-04340-s001.zip › molecules-3149938-supplementary.pdf]

Supplementary Materials

Conformational Analysis and Organocatalytic Activity of Helical Stapled Peptides Containing α-Carbocyclic α,α-Disubstituted α-Amino Acids

Akihiro Iyoshi [1], Atsushi Ueda [1,*], Tomohiro Umeno [1], Takuma Kato [2],
Kazuhiro Hirayama [1], Mitsunobu Doi [2] and Masakazu Tanaka [1,*]

[1] Graduate School of Biomedical Sciences, Nagasaki University, 1-14 Bunkyo-machi, Nagasaki 852-8521, Japan

[2] Faculty of Pharmacy, Osaka Medical and Pharmaceutical University, Osaka 569-1094, Japan

* Correspondence: aueda@nagasaki-u.ac.jp (A.U.); matanaka@nagasaki-u.ac.jp (M.T.)

Table of Contents

1. X-ray crystallographic analysis of peptides **2a** and **2e** ······ S2
2. CD spectra of peptides **1a**–**1e** and **2a**–**2e** ······ S8
3. NMR spectra ······ S9
4. HPLC Charts ······ S22

1. X-ray crystallographic analysis of peptides 2a and 2e.

1.1. X-Ray crystallographic data of peptide 2a.

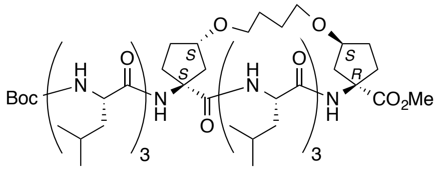

Table S1. Crystal and diffraction parameters of peptide **2a**.

Empirical formula	$C_{58}H_{102}N_8O_{13}$, CH_4O, $2(H_2O)$
Formula weight	1187.55
Crystal dimensions (mm)	0.20 x 0.18 x 0.04
Data collection temp. (K)	93
Crystal system	monoclinic
Lattice parameters	
a, b, c (Å)	11.020, 26.147, 12.427
α, β, γ (°)	90, 102.58, 90
V (Å^3)	3490.9
Space group	$P\,2_1$
Z value	2
D calc (g/cm^3)	1.130
μ (MoKα)(cm^{-1})	0.082
No. of observations	5902
No. of variable	779
R_1 ($I > 2\sigma$)	0.0903
wR_2	0.2440
Crystallizing solvent	MeOH/H_2O

Table S2. Intra- and intermolecular H-bond parameters for peptide **2a**.

Donor D–H	Acceptor A	Distance (Å) D···A	Angle (°) D–H···A	Symmetry operations
N_4-H	O_0	2.95	153.1	x,y,z
N_5-H	O_1	3.05	160.9	x,y,z
N_6-H	O_2	2.97	162.5	x,y,z
N_7-H	O_3	2.95	148.3	x,y,z
N_8-H	O_4	2.95	101.4[a]	x,y,z
N_3-H	O_{W1}	2.91	166.2	x,y,z
O_{W1}-H_1	O_M	2.90	175.2	x,y,z
O_{W2}-H_1	O_6	2.75	171.2	x,y,z
O_{W2}-H_2	O_8	2.79	172.4	x,y,z
N_1-H	$O_{6'}$	2.89	173.4	$2-x,-1/2+y,1-z$
N_2-H	$O_{W2'}$	2.97	173.2	$2-x,-1/2+y,1-z$
$O_{M'}$-H	O_5	2.79	170.1	$2-x,-1/2+y,1-z$

O_{W1}-H_2	$O_{W2'}$	2.80	169.4	2–x,1/2+y,1–z

[a]The angle N_8-H···O_4 is too small for a hydrogen bond.

Table S3. Selected torsion angles ω, ϕ, ψ, and χ (°) for peptide **2a**, as determined by an X-ray crystallographic analysis.

ω0	–168.7	ϕ5	–61.1	χ1	–177.1
ϕ1	–69.9	ψ5	–45.7	χ2	–61.7
ψ1	–50.3	ω5	–179.4	χ3	–66.7
ω1	–175.3	ϕ6	–58.8	χ4	–99.4
ϕ2	–65.4	ψ6	–36.9	χ4′	84.3
ψ2	–41.8	ω6	–175.3	χ5	–64.6
ω2	177.5	ϕ7	–98.6	χ6	–171.6
ϕ3	–69.4	ψ7	5.7	χ7	–84.8
ψ3	–32.7	ω7	–171.7	χ8	135.1
ω3	171.6	ϕ8	–63.7	χ8′	–112.0
ϕ4	–54.3	ψ8	154.9		
ψ4	–49.3	ω8	175.6		
ω4	–178.1				

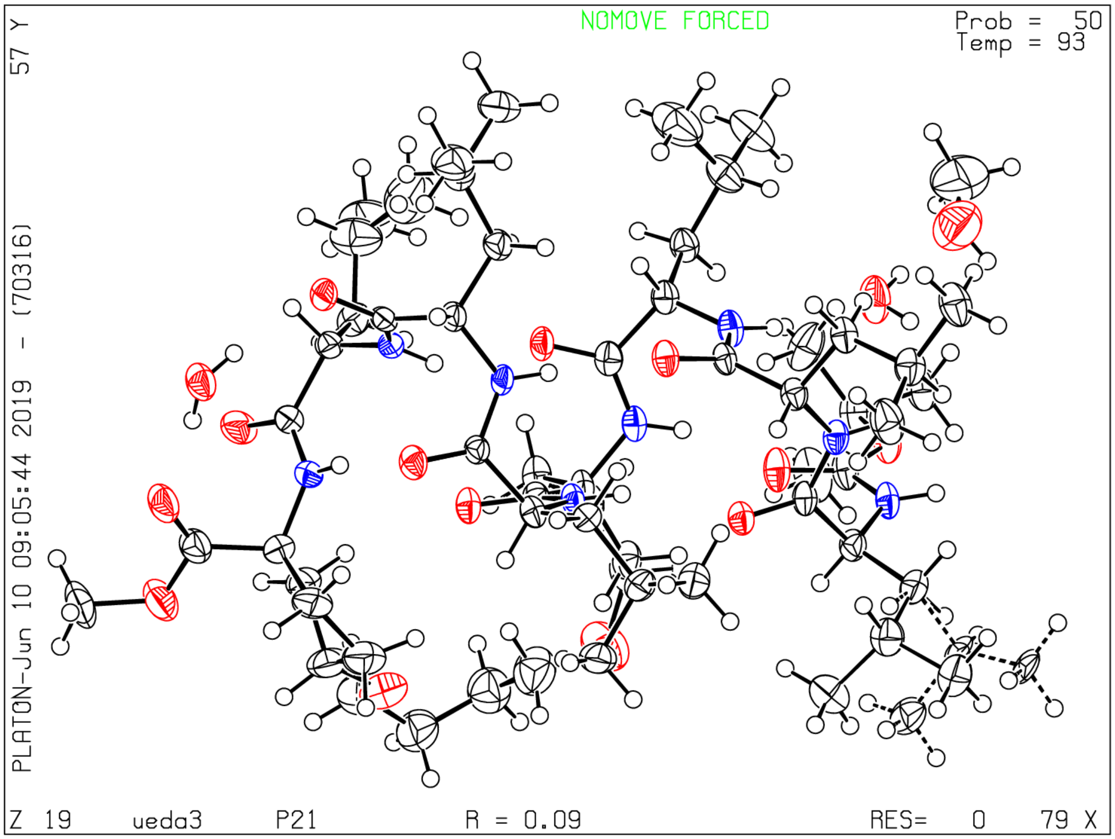

Figure S1. ORTEP drawing of peptide **2a**.

1.2. X-Ray crystallographic data of peptide 2e

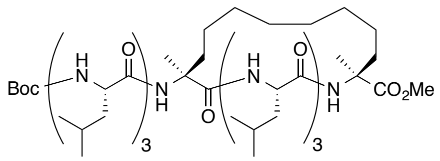

Table S4. Crystal and diffraction parameters of peptide **2e**.

Empirical formula	$C_{56}H_{102}N_8O_{11}$
Formula weight	1063.45
Crystal dimensions (mm)	0.32 x 0.137 x 0.093
Data collection temp. (K)	93
Crystal system	orthorhombic
Lattice parameters	
a, *b*, *c* (Å)	12.738, 27.841, 53.647
α, β, γ (°)	90, 90, 90
V (Å^3)	19025.4
Space group	$P\ 2_12_12_1$
Z value	12
D calc (g/cm^3)	1.114
μ (CuKα)(cm^{-1})	0.620
No. of observations	28705
No. of variable	2211
R_1 ($I > 2\sigma$)	0.0490
wR_2	0.1290
Crystallizing solvent	MeOH/EtOAc/*n*-hexane

Table S5. Intra- and intermolecular H-bond parameters for peptide **2e**.

Donor D–H	Acceptor A	Distance (Å) D···A	Angle (°) D–H···A	Symmetry operations
Mol A				
N_3-H	O_0	3.11	131.2	*x,y,z*
N_4-H	O_0	3.01	167.1	*x,y,z*
N_5-H	O_1	3.02	154.4	*x,y,z*
N_6-H	O_2	3.94	121.0[b]	*x,y,z*
N_6-H	O_3	2.98	154.3	*x,y,z*
N_7-H	O_3	3.80[a]	131.8	*x,y,z*
N_7-H	O_4	2.96	151.3	*x,y,z*
N_8-H	O_4	2.99	172.5	*x,y,z*
N_8-H	O_5	4.43[a]	110.2[b]	*x,y,z*
N_1-H	$O_{6'}$	2.91	149.3	*x*,–1+*y*,*z*
N_2-H	$O_{7'}$	2.88	159.5	*x*,–1+*y*,*z*
Mol B				

N_3-H	O_0	3.05	133.3	*x,y,z*
N_4-H	O_0	2.96	169.3	*x,y,z*
N_5-H	O_1	3.03	158.0	*x,y,z*
N_6-H	O_2	4.13[a]	128.8[b]	*x,y,z*
N_6-H	O_3	2.93	146.6	*x,y,z*
N_7-H	O_3	3.70[a]	124.8[b]	*x,y,z*
N_7-H	O_4	2.92	156.0	*x,y,z*
N_8-H	O_4	3.53	125.9[b]	*x,y,z*
N_8-H	O_5	4.09[a]	155.0	*x,y,z*
N_1-H	$O_{6'}$	2.85	169.8	*x,y,z*
N_2-H	$O_{7'}$	2.94	160.1	*x,y,z*
Mol C (major)				
N_3-H	O_0	3.14	129.9[b]	*x,y,z*
N_4-H	O_0	3.00	166.3	*x,y,z*
N_5-H	O_1	3.02	153.3	*x,y,z*
N_6-H	O_2	3.89[a]	122.3[b]	*x,y,z*
N_6-H	O_3	2.94	151.4	*x,y,z*
N_7-H	O_3	3.72[a]	128.7[b]	*x,y,z*
N_7-H	O_4	2.95	152.4	*x,y,z*
N_8-H	O_4	3.12	177.5	*x,y,z*
N_8-H	O_5	4.57[a]	106.5[b]	*x,y,z*
N_1-H	$O_{6'}$	2.87	164.2	–1–*x*,–1/2+*y*,1.5–*z*
N_2-H	$O_{7'}$	2.96	164.0	–1–*x*,–1/2+*y*,1.5–*z*

[a]These lengths are too long for a hydrogen bond. [b]These angles are too small for a hydrogen bond.

Table S6. Selected torsion angles ω, ϕ, ψ, and χ (°) for peptide **2e**, as determined by an X-ray crystallographic analysis.

Mol A		Mol B		Mol C	
ω0	–175.7	ω0	–170.5	ω0	–171.1
ϕ1	–65.3	ϕ1	–58.7	ϕ1	–64.1
ψ1	–35.4	ψ1	–36.4	ψ1	–36.3
ω1	–178.9	ω1	–176.1	ω1	–177.8
ϕ2	–65.5	ϕ2	–70.6	ϕ2	–67.9
ψ2	–25.5	ψ2	–22.5	ψ2	–25.2
ω2	173.1	ω2	169.2	ω2	171.4
ϕ3	–80.5	ϕ3	–84.1	ϕ3	–80.6
ψ3	–44.4	ψ3	–41.9	ψ3	–42.0
ω3	179.4	ω3	179.5	ω3	178.8
ϕ4	–51.2	ϕ4	–50.1	ϕ4	–51.1
ψ4	–43.0	ψ4	–46.2	ψ4	–42.9
ω4	–171.8	ω4	–172.5	ω4	–173.2
ϕ5	–75.6	ϕ5	–70.1	ϕ5	–76.9
ψ5	–3.4	ψ5	–12.3	ψ5	–3.0

ω5	167.6	ω5	–171.0	ω5	165.7
ϕ6	–83.2	ϕ6	–78.7	ϕ6	–81.6
ψ6	–16.4	ψ6	–16.2	ψ6	–18.5
ω6	179.6	ω6	–174.2	ω6	–175.4
ϕ7	–98.5	ϕ7	–97.8	ϕ7	–92.4
ψ7	–54.4	ψ7	–19.0	ψ7	–57.2
ω7	–171.0	ω7	–172.5	ω7	–176.2
ϕ8	–90.0	ϕ8	–172.6	ϕ8	–93.1
ψ8	–48.3	ψ8	9.1	ψ8	–36.9
ω8	–173.7	ω8	171.8	ω8	–164.1
χ1	–60.0	χ1	–71.4	χ1	–74.3
χ2	–62.7	χ2	–61.0	χ2	–62.8
χ3	–55.0	χ3	–61.4	χ3	–55.9
χ4	–178.6	χ4	–178.1	χ4	–179.1
χ5	–65.1	χ5	–67.0	χ5	–67.0
χ6	–56.2	χ6	–58.3	χ6	–59.2
χ7	–81.8	χ7	–61.8	χ7	–87.3
χ8	173.9	χ8	46.6	χ8	167.2

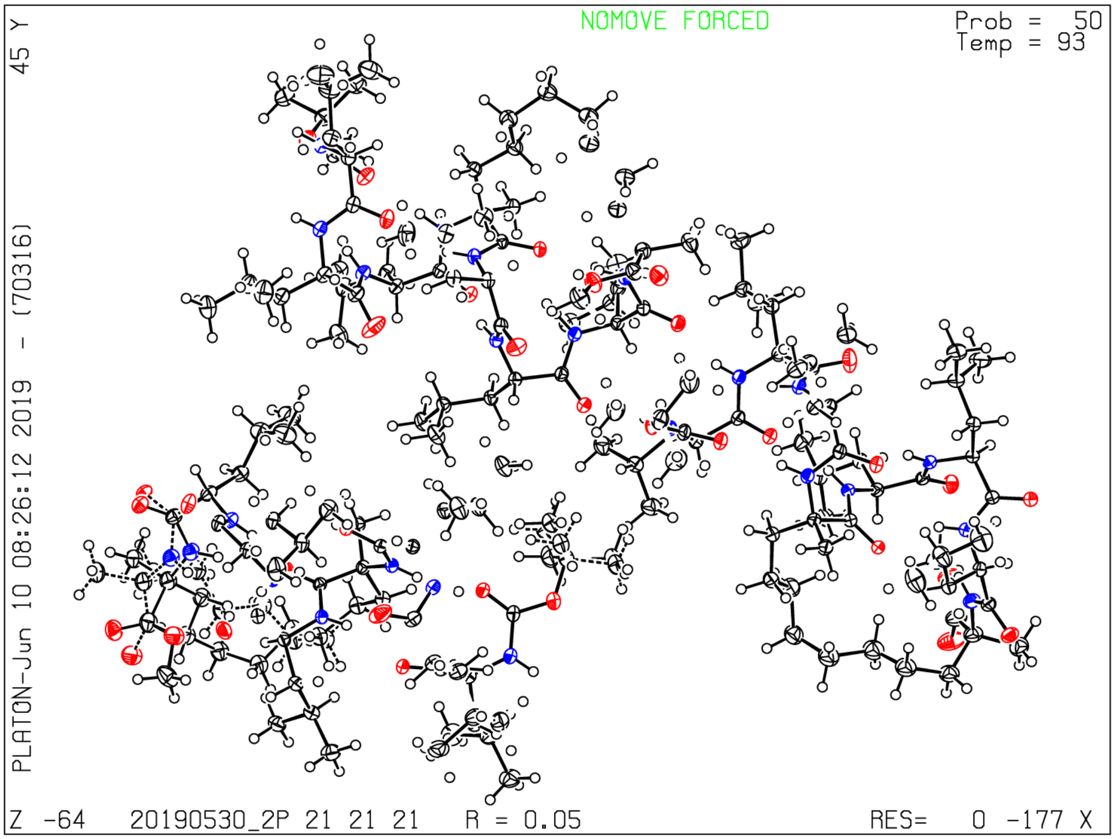

Figure S2. ORTEP drawing of peptide **2e**.

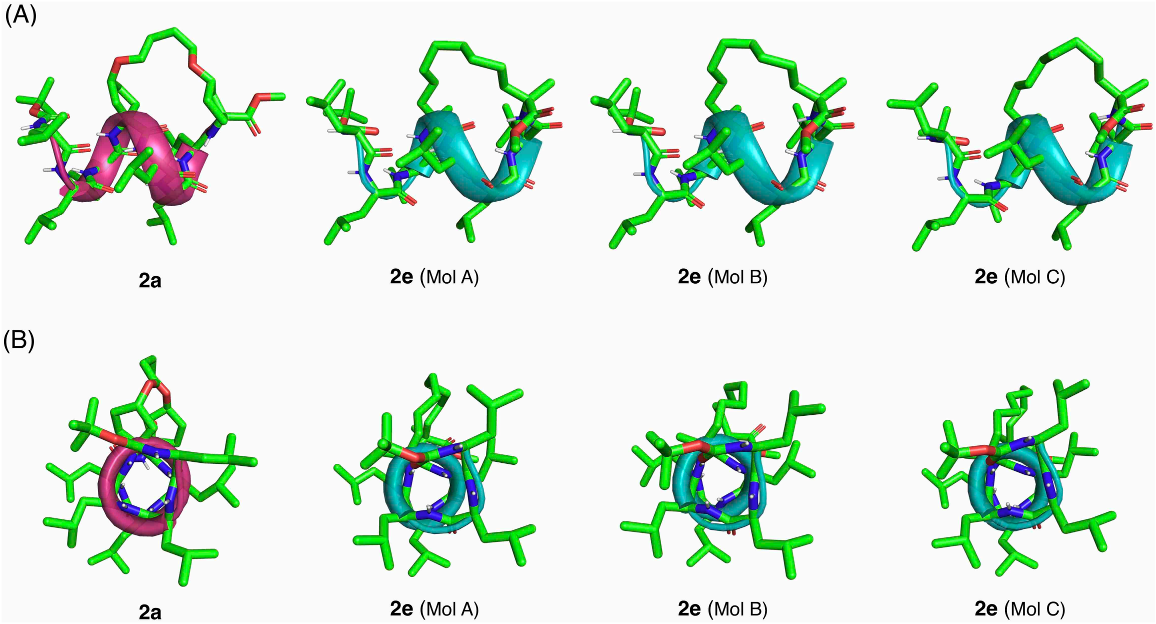

Figure S3. X-ray crystallographic structures of peptides **2a**, **2e** (Mol A), **2e** (Mol B) , **2e** (Mol C). (A) A view perpendicular to the helical axis and (B) a view along the helical axis.

2. CD spectra of peptides 1a–1e and 2a–2e.

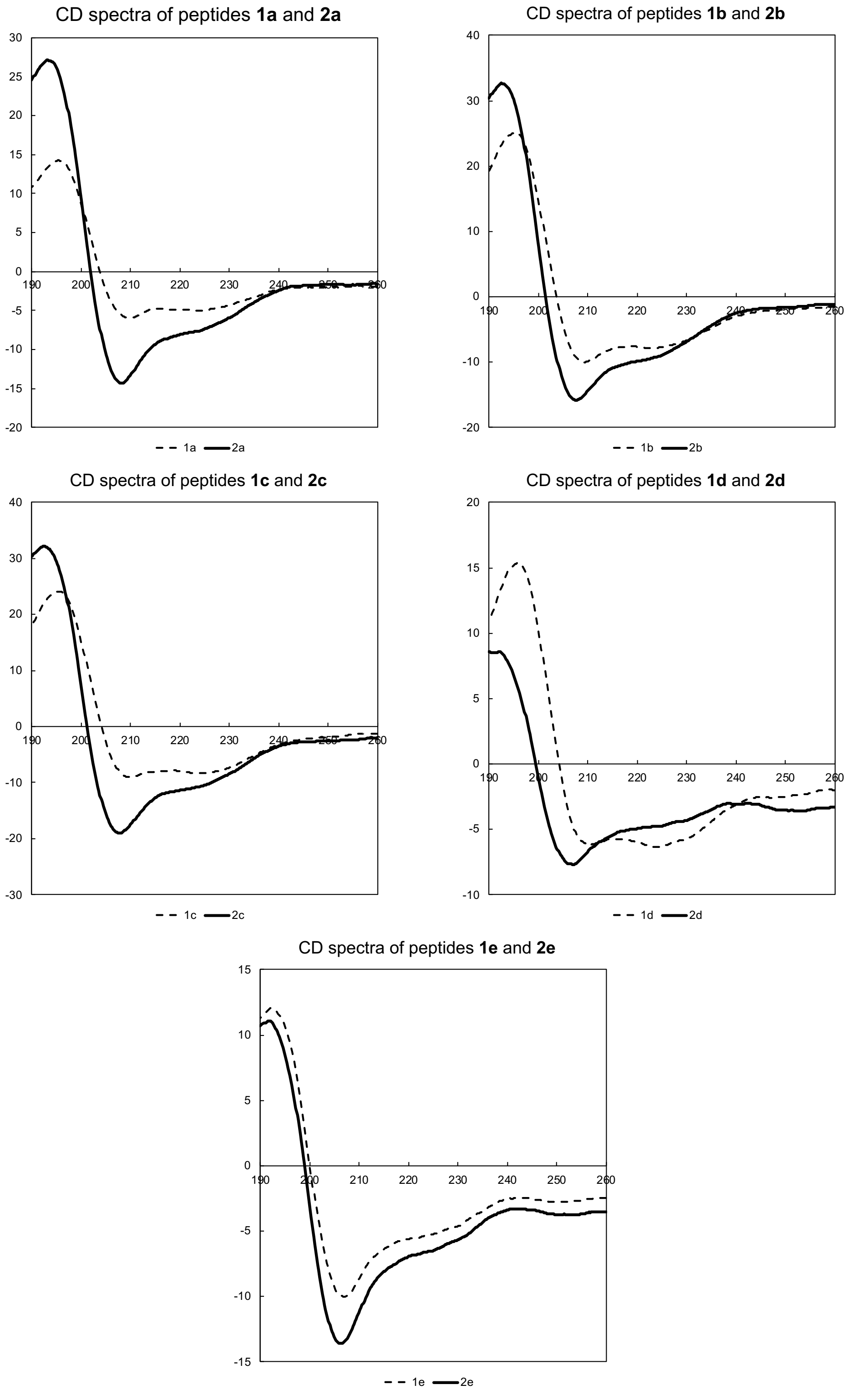

Figure S4. CD spectra of unstapled peptides **1a**–**1e** and stapled peptides **2a**–**2e** in 10% MeOH/H_2O (concentration: 0.050 mM).

3. NMR spectra.

^{1}H NMR of **2a** (500 MHz, $CDCl_3$)

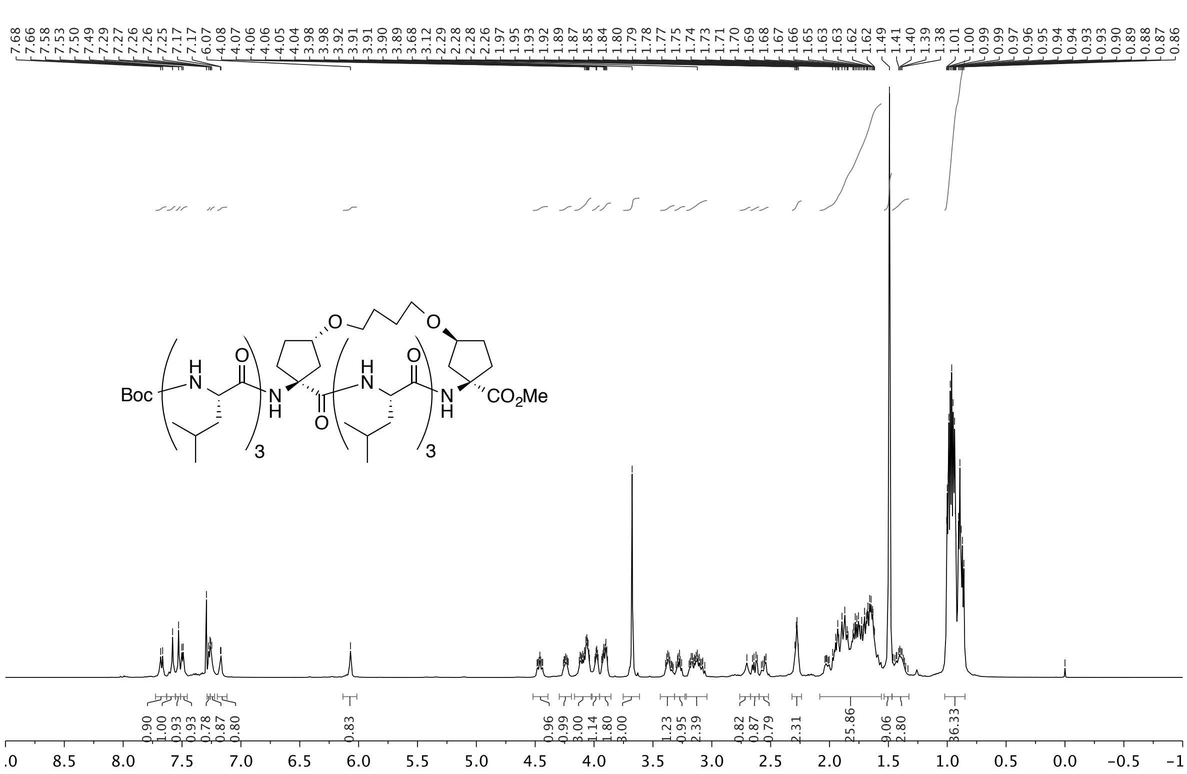

^{13}C NMR of **2a** (125 MHz, $CDCl_3$)

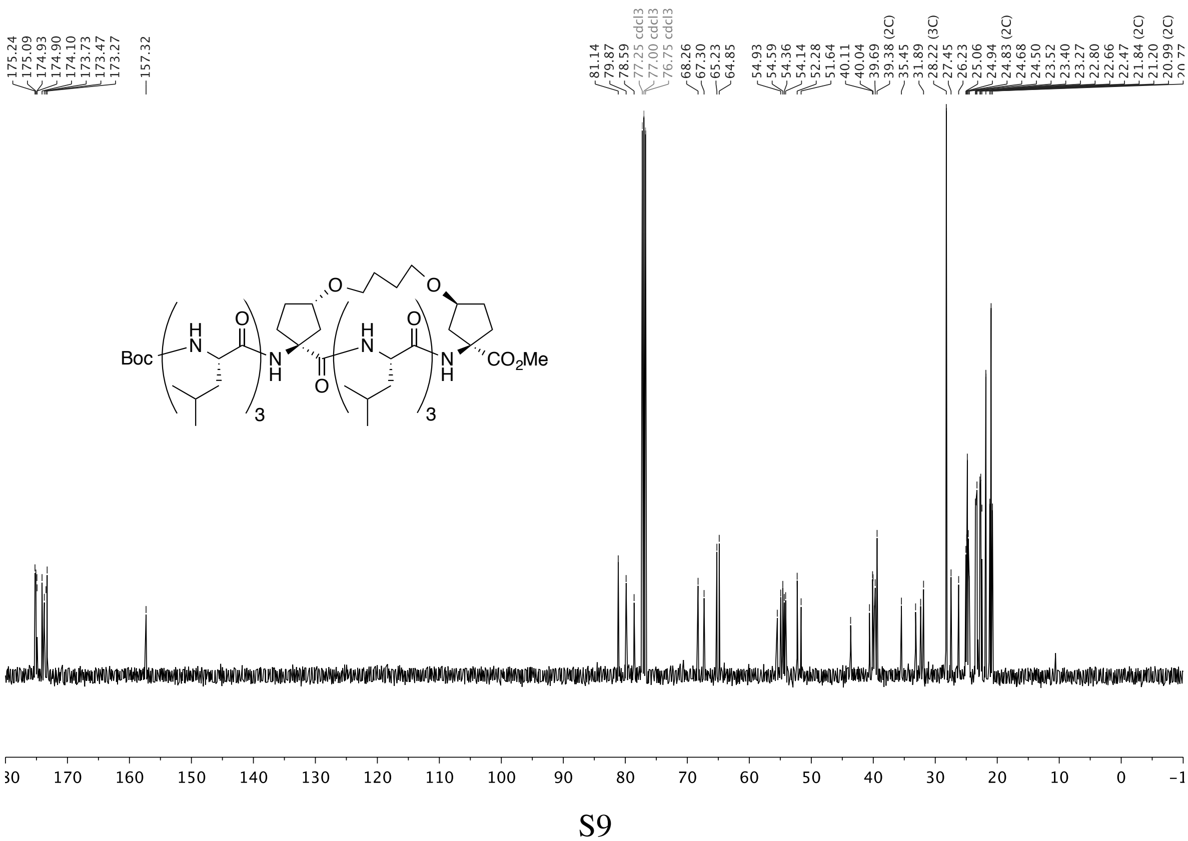

^{1}H NMR of **2b** (500 MHz, $CDCl_3$)

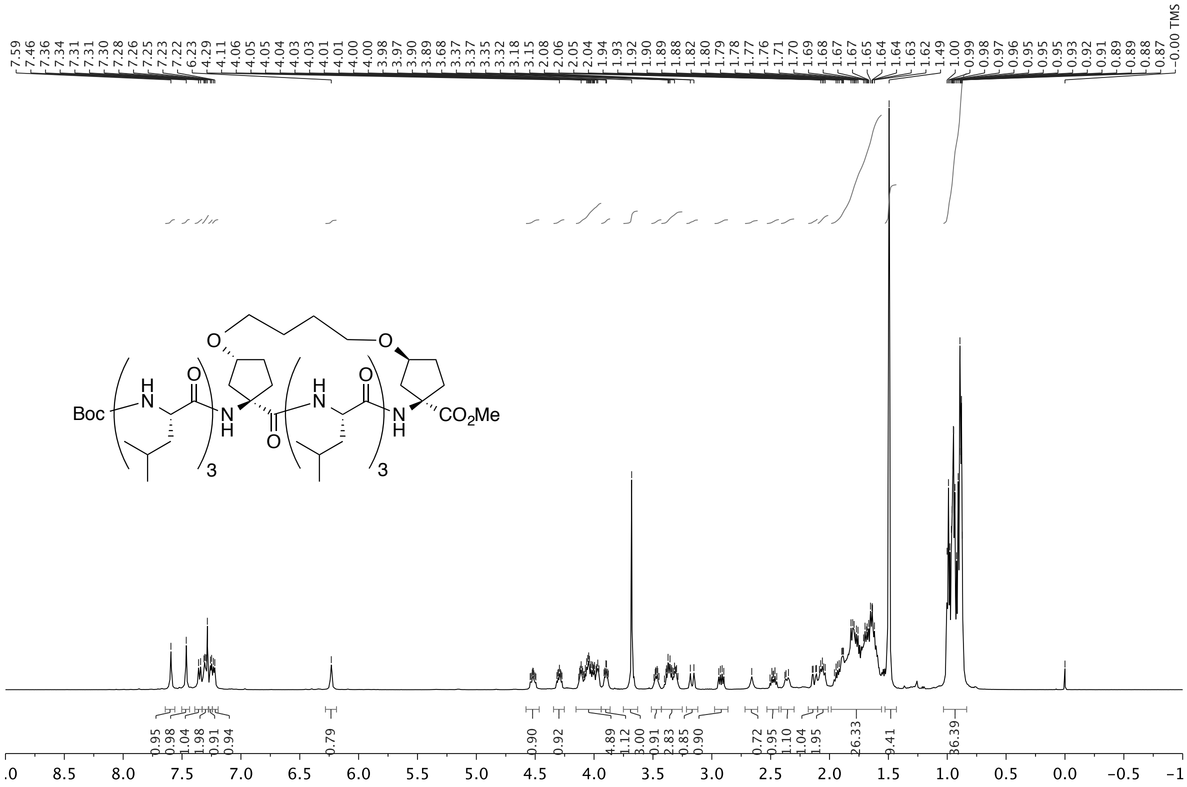

^{13}C NMR of **2b** (125 MHz, $CDCl_3$)

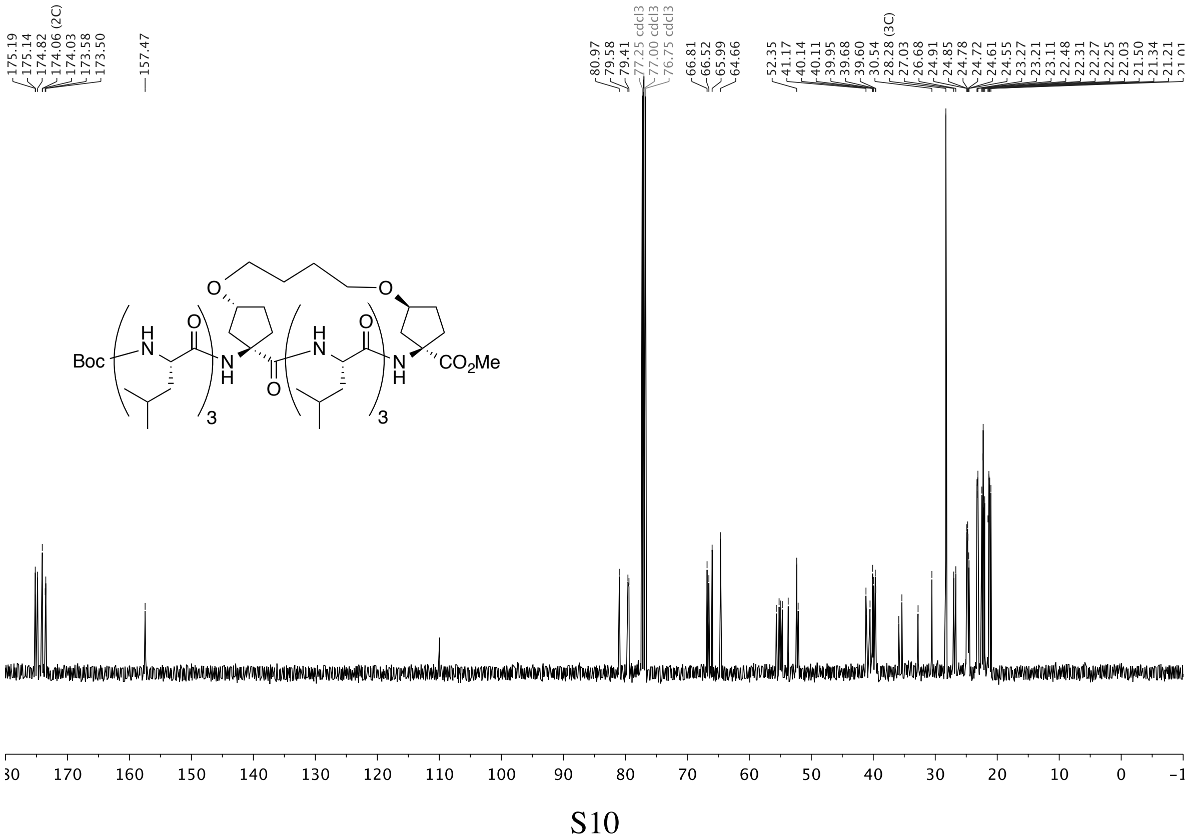

^{1}H NMR of **2c** (500 MHz, $CDCl_3$)

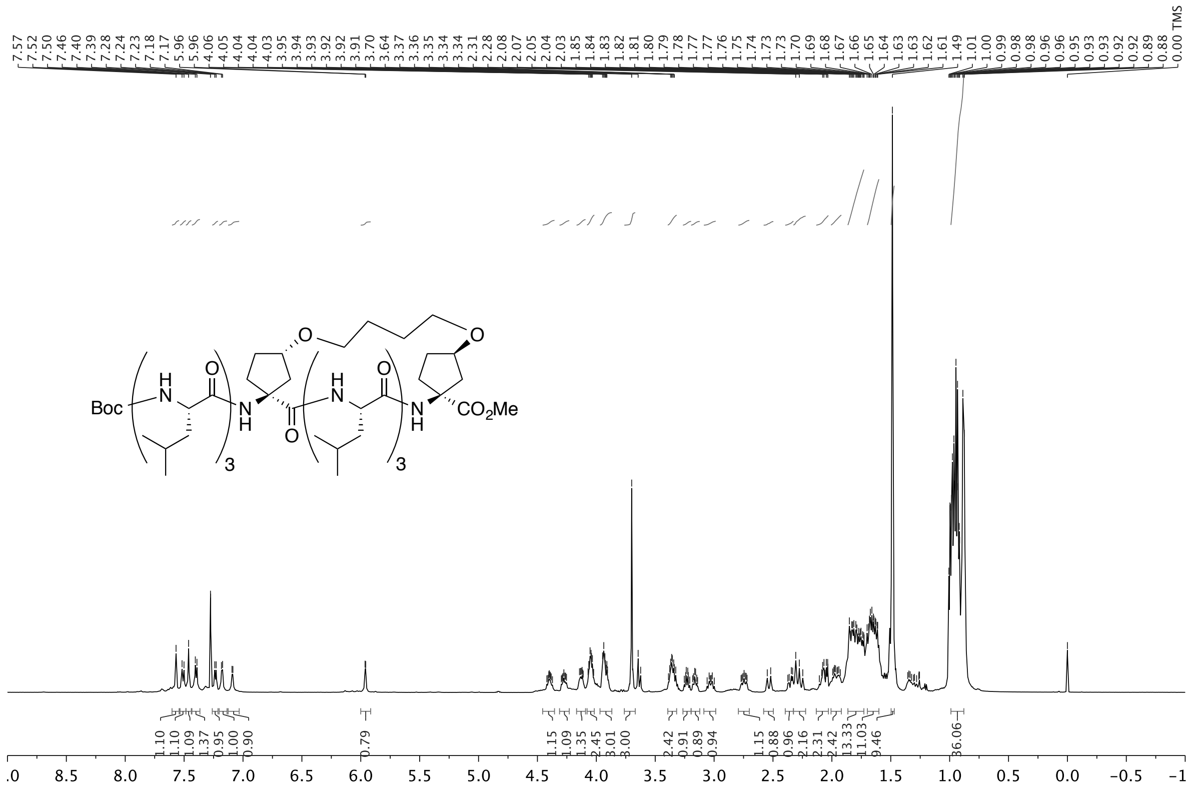

^{13}C NMR of **2c** (125 MHz, $CDCl_3$)

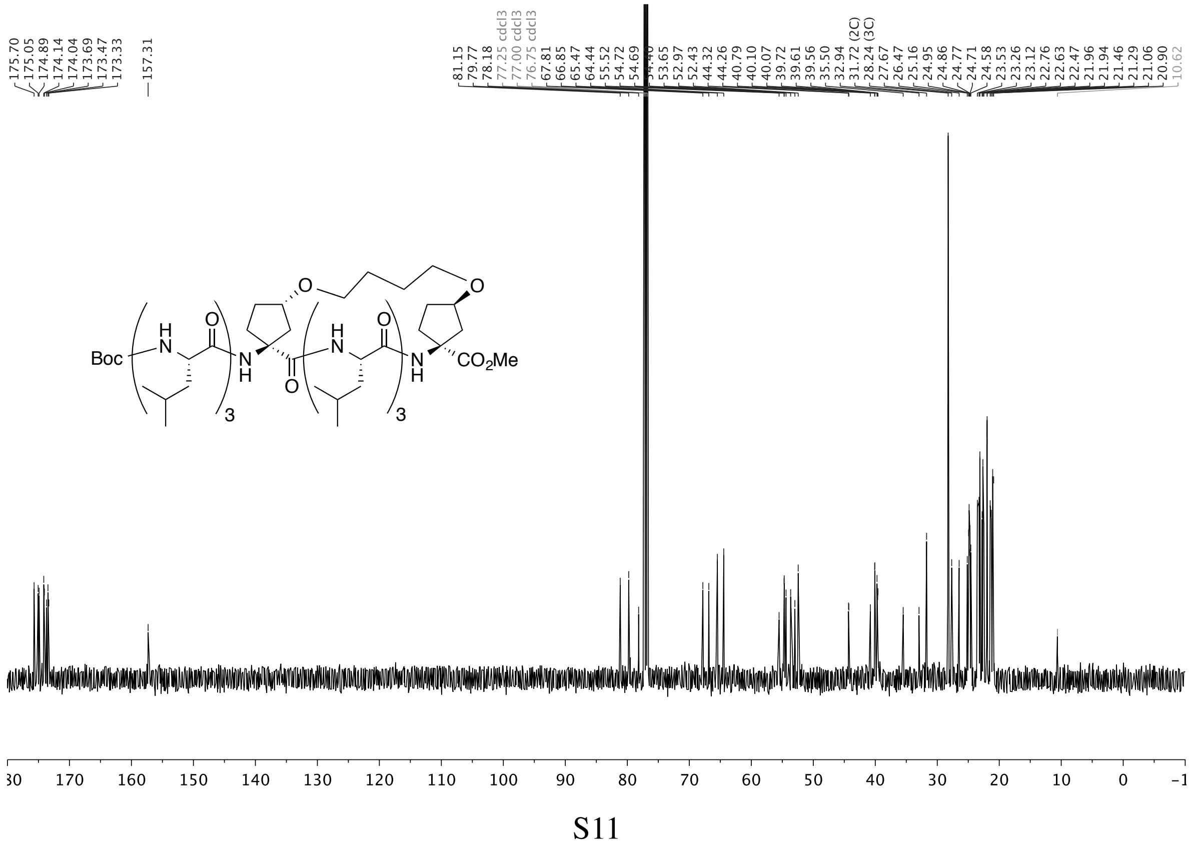

^{1}H NMR of **2d** (500 MHz, $CDCl_3$)

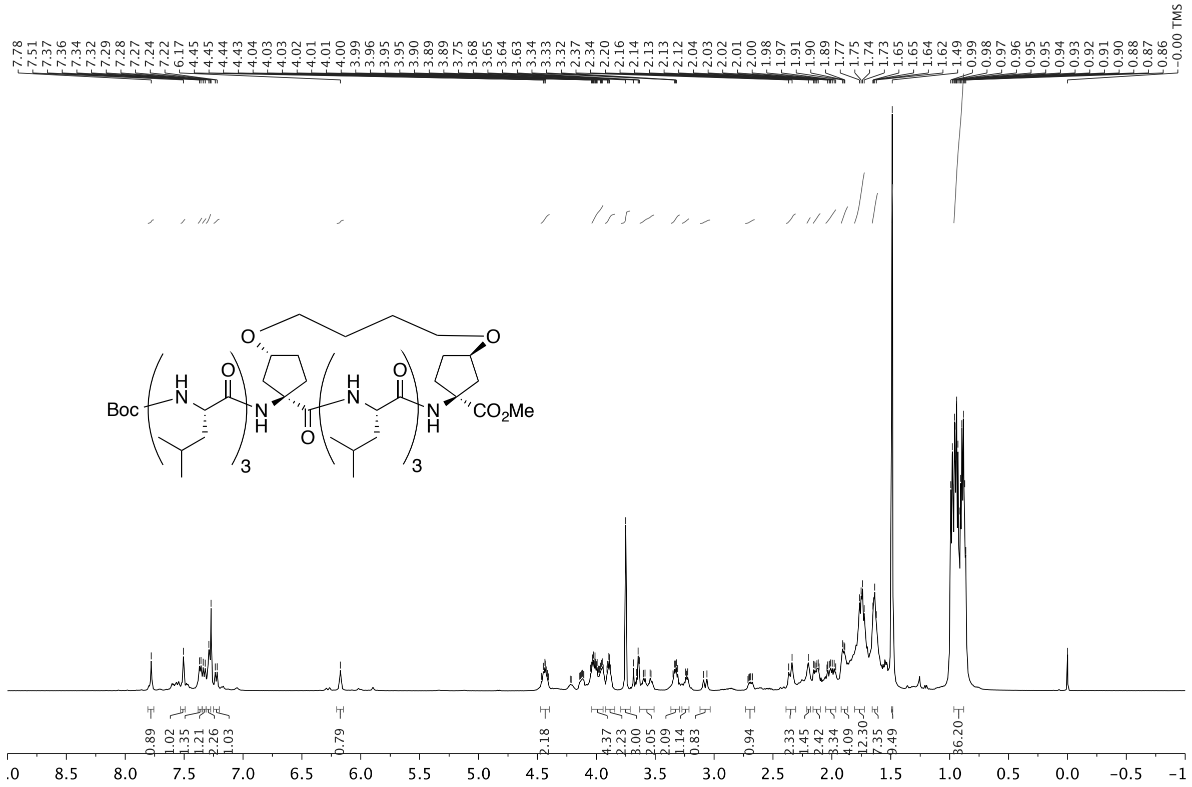

^{13}C NMR of **2d** (125 MHz, $CDCl_3$)

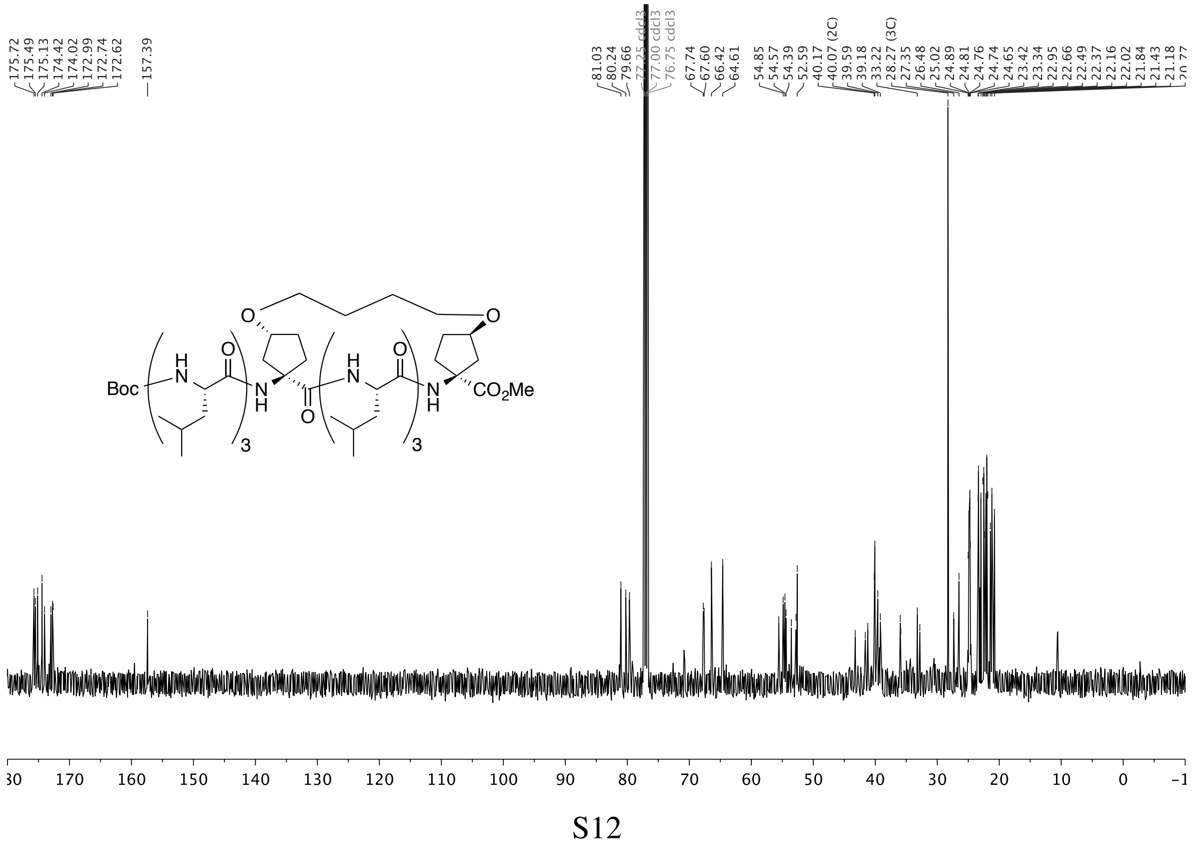

^{1}H NMR of **2e** (500 MHz, $CDCl_3$)

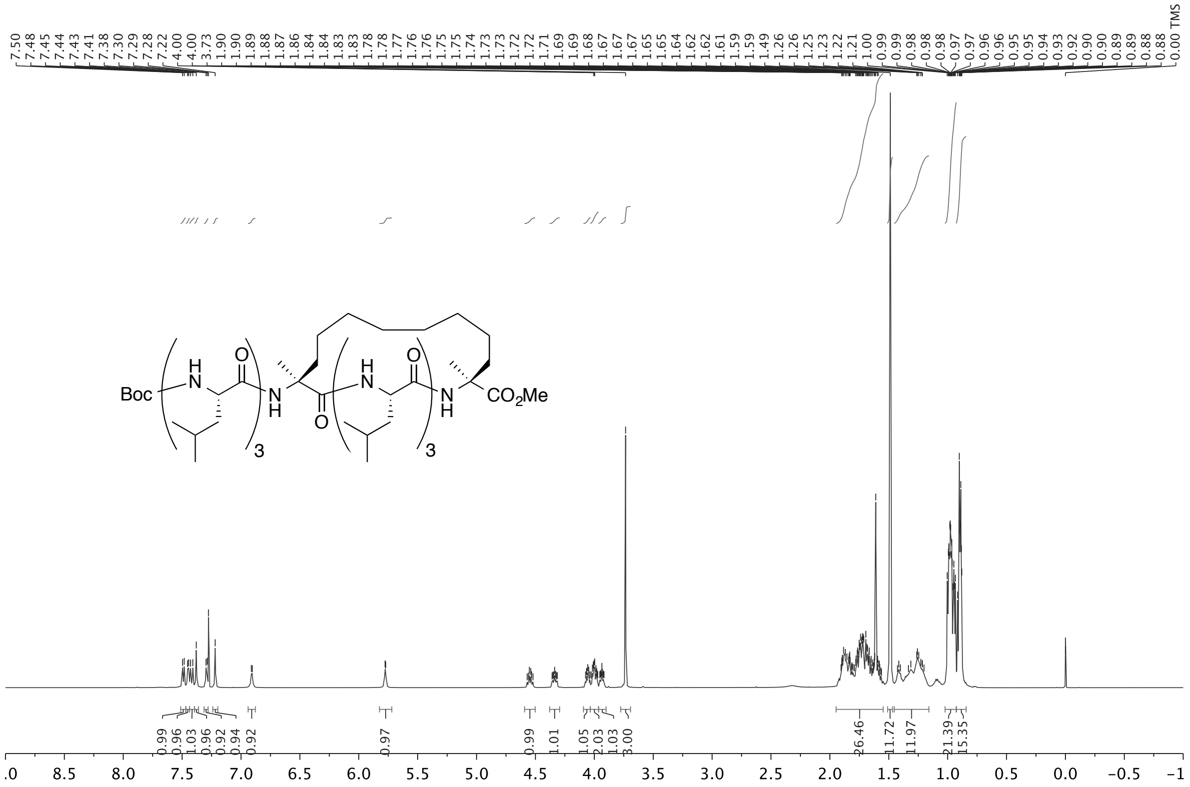

^{13}C NMR of **2e** (125 MHz, $CDCl_3$)

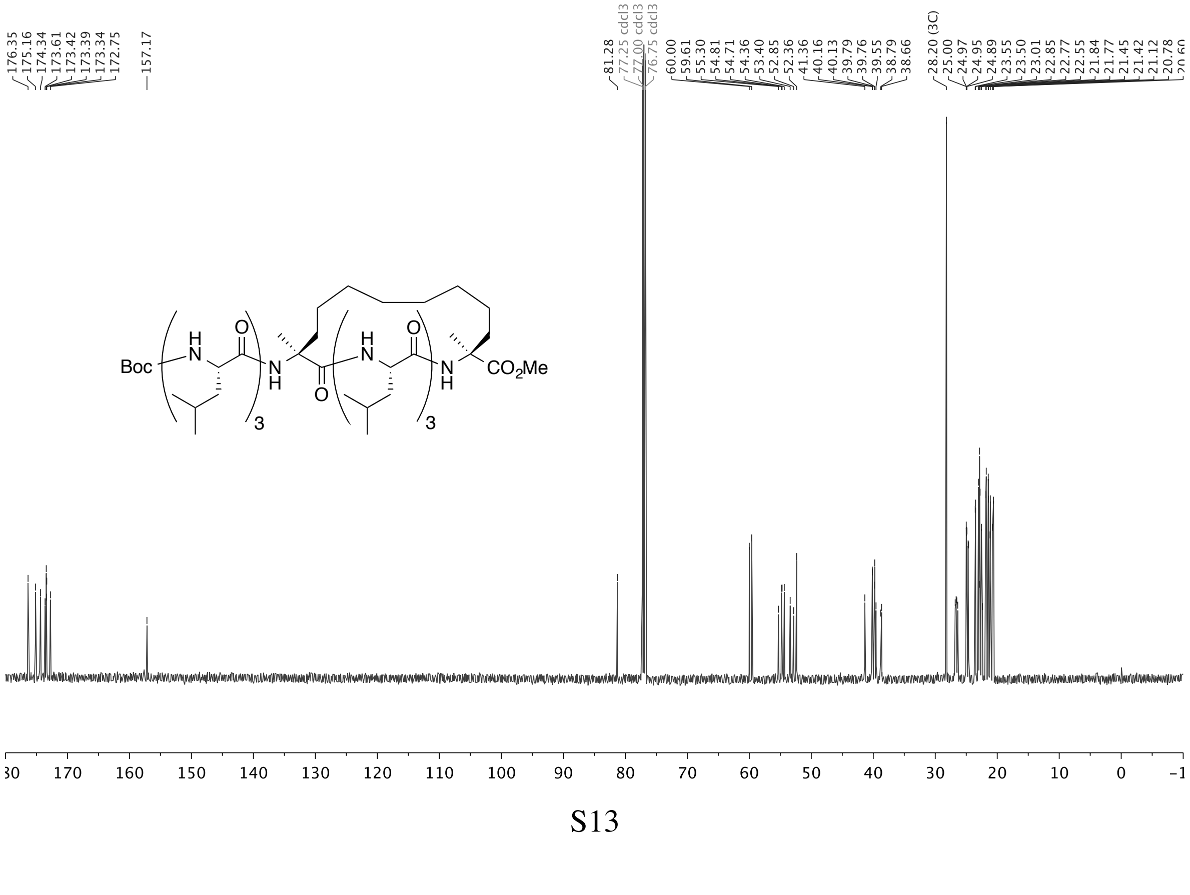

^{1}H NMR of **1a′** (500 MHz, $CDCl_3$)

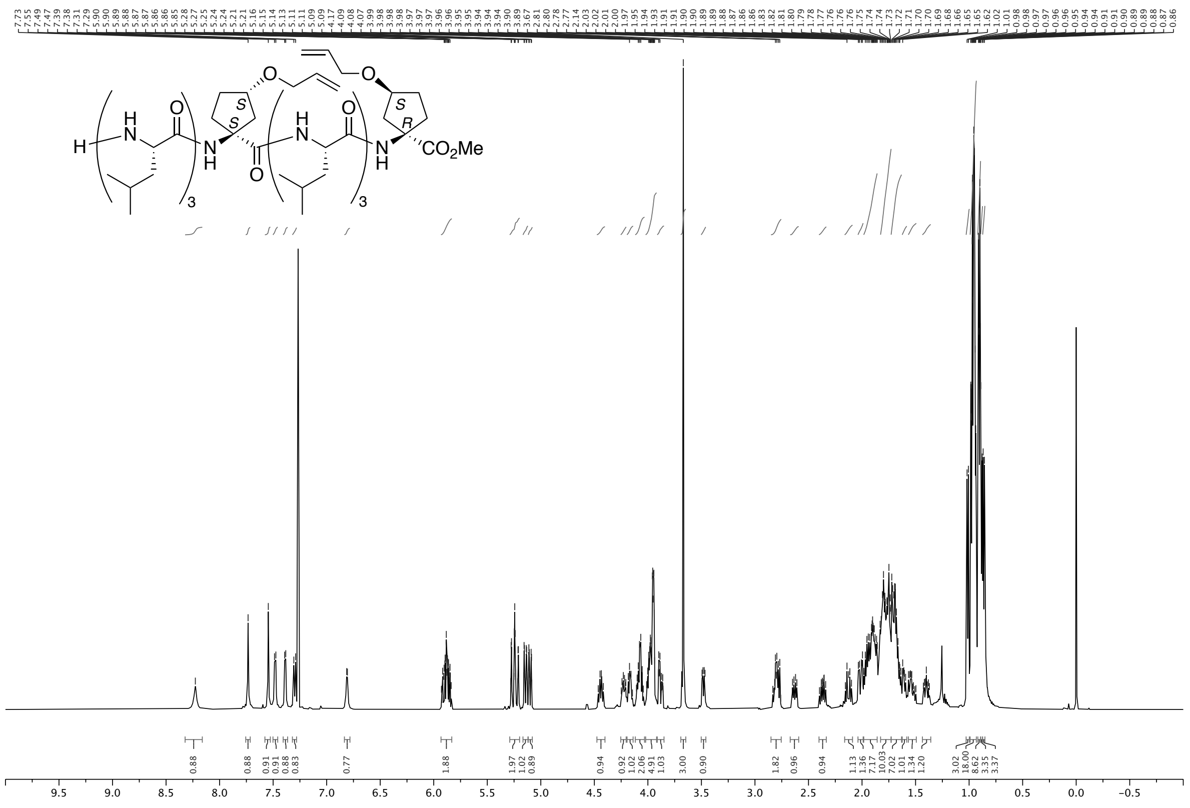

^{1}H NMR of **1b′** (500 MHz, $CDCl_3$)

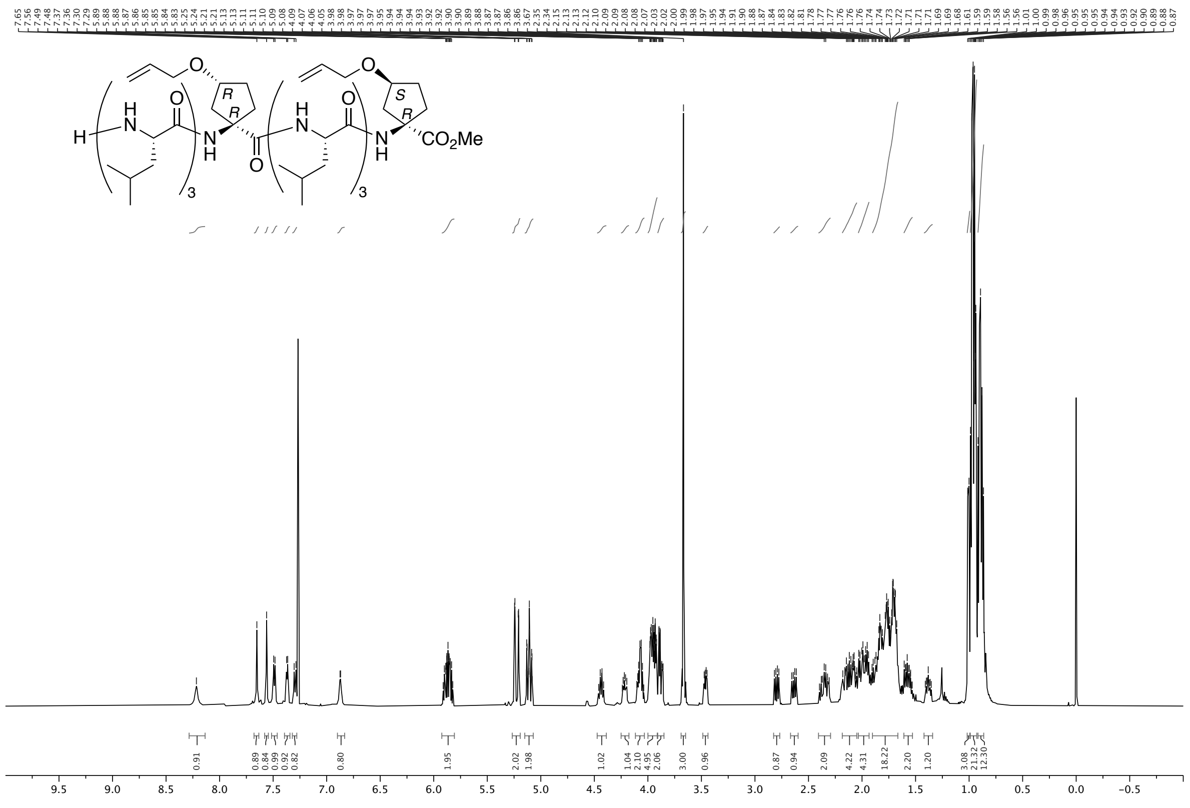

^{1}H NMR of **1c′** (500 MHz, $CDCl_3$)

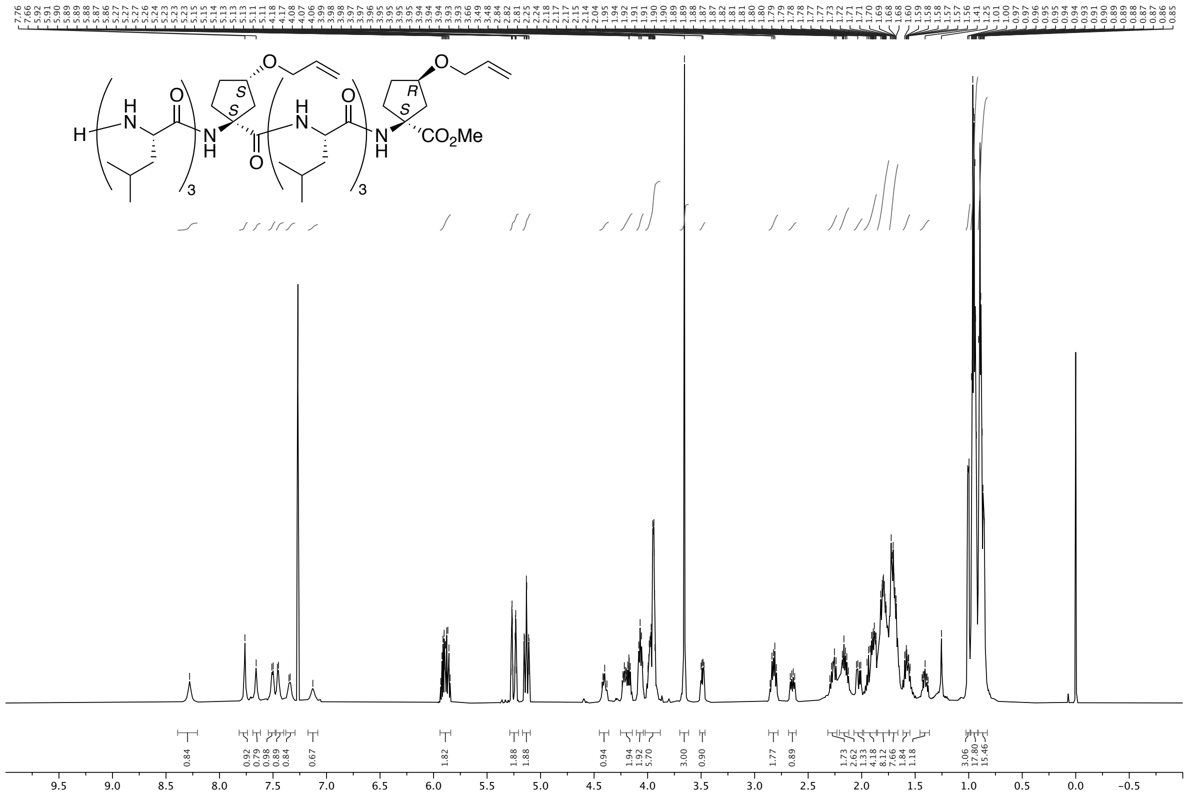

^{1}H NMR of **1d′** (500 MHz, $CDCl_3$)

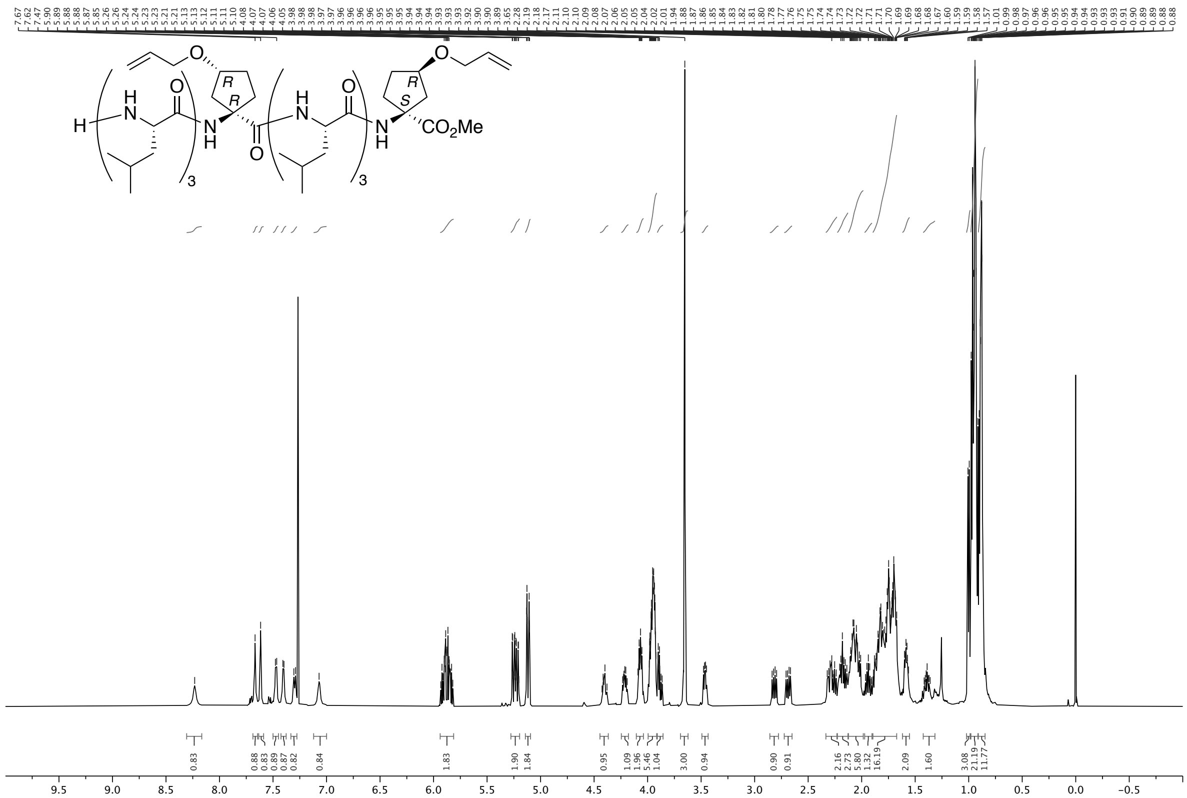

^{1}H NMR of **1e′** (500 MHz, $CDCl_3$)

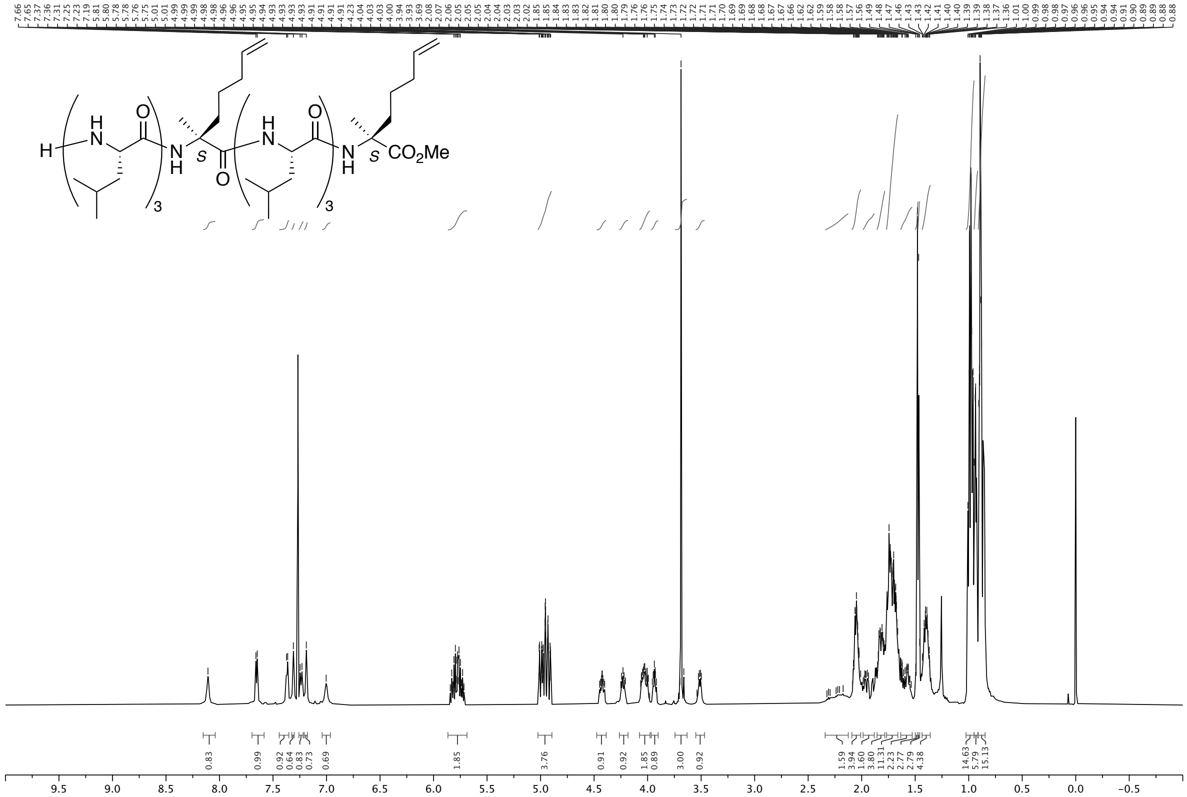

^{1}H NMR of **2a′** (500 MHz, $CDCl_3$)

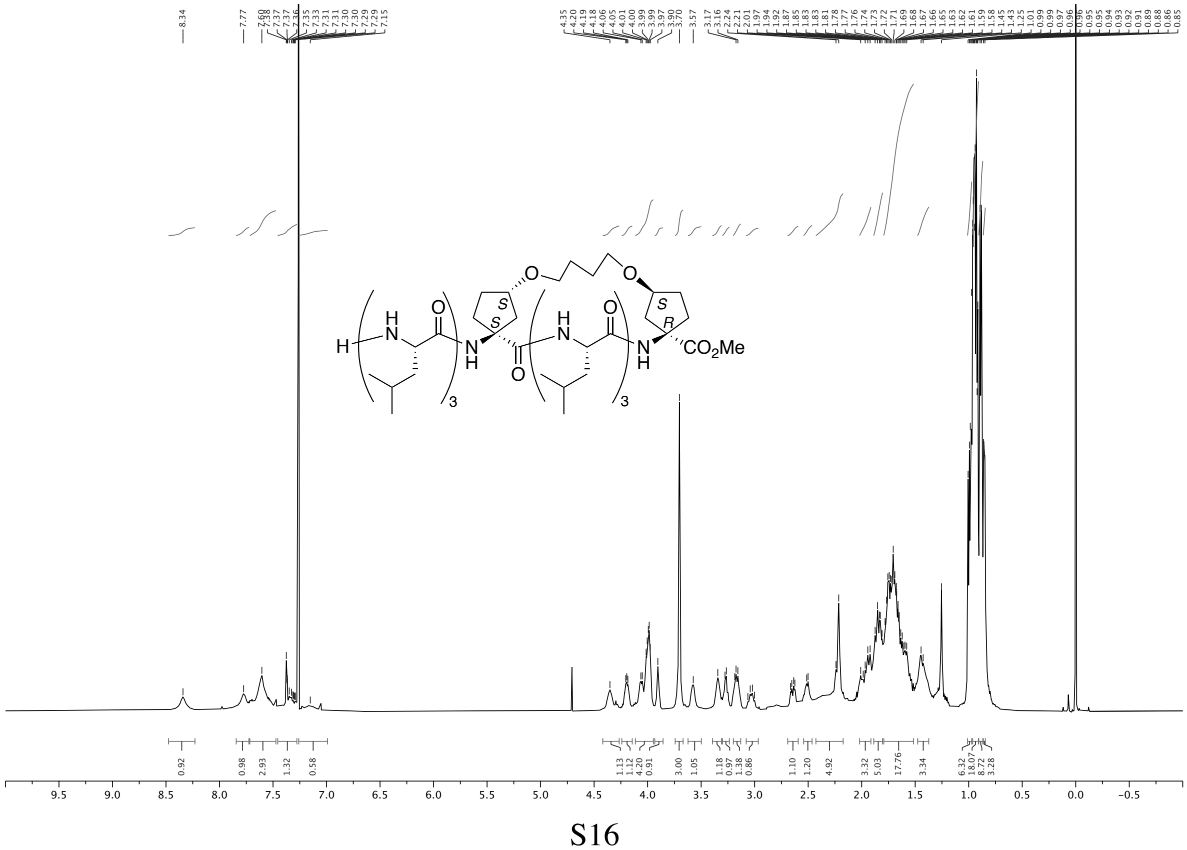

^{1}H NMR of **2b′** (500 MHz, $CDCl_3$)

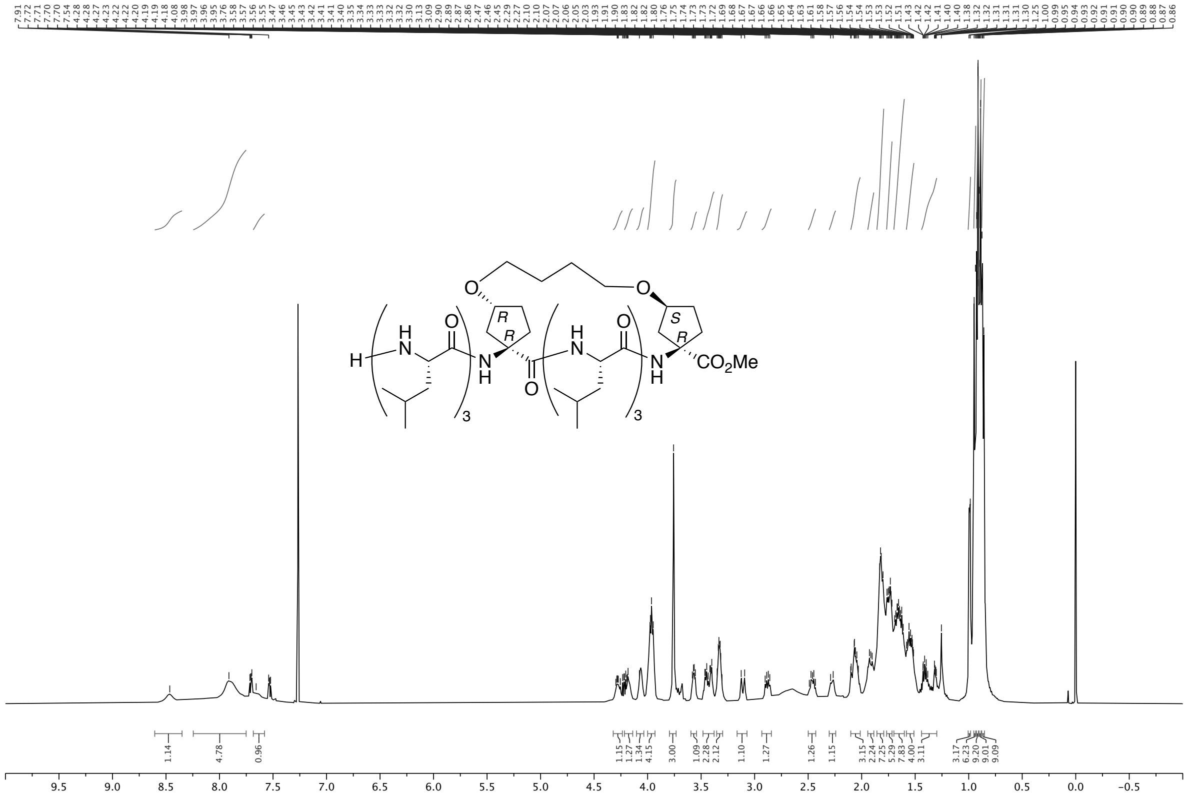

^{1}H NMR of **2c′** (500 MHz, $CDCl_3$)

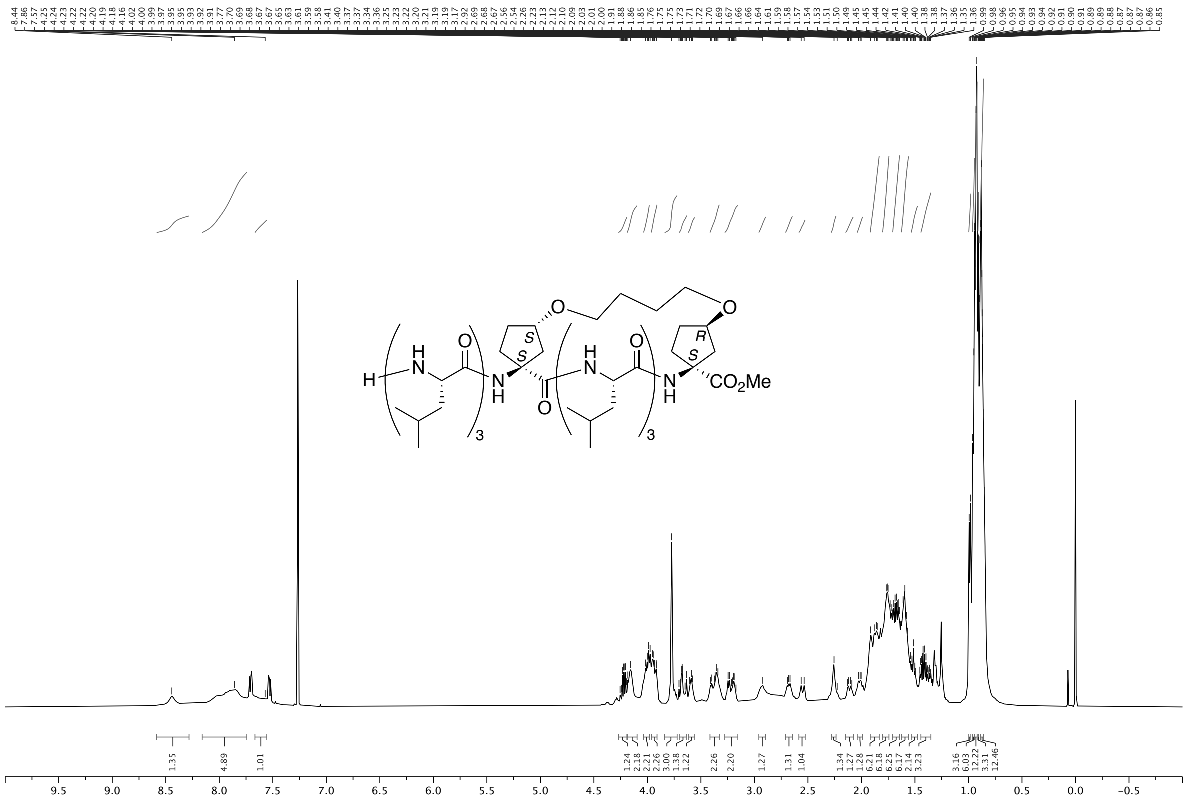

^{1}H NMR of **2d′** (500 MHz, $CDCl_3$)

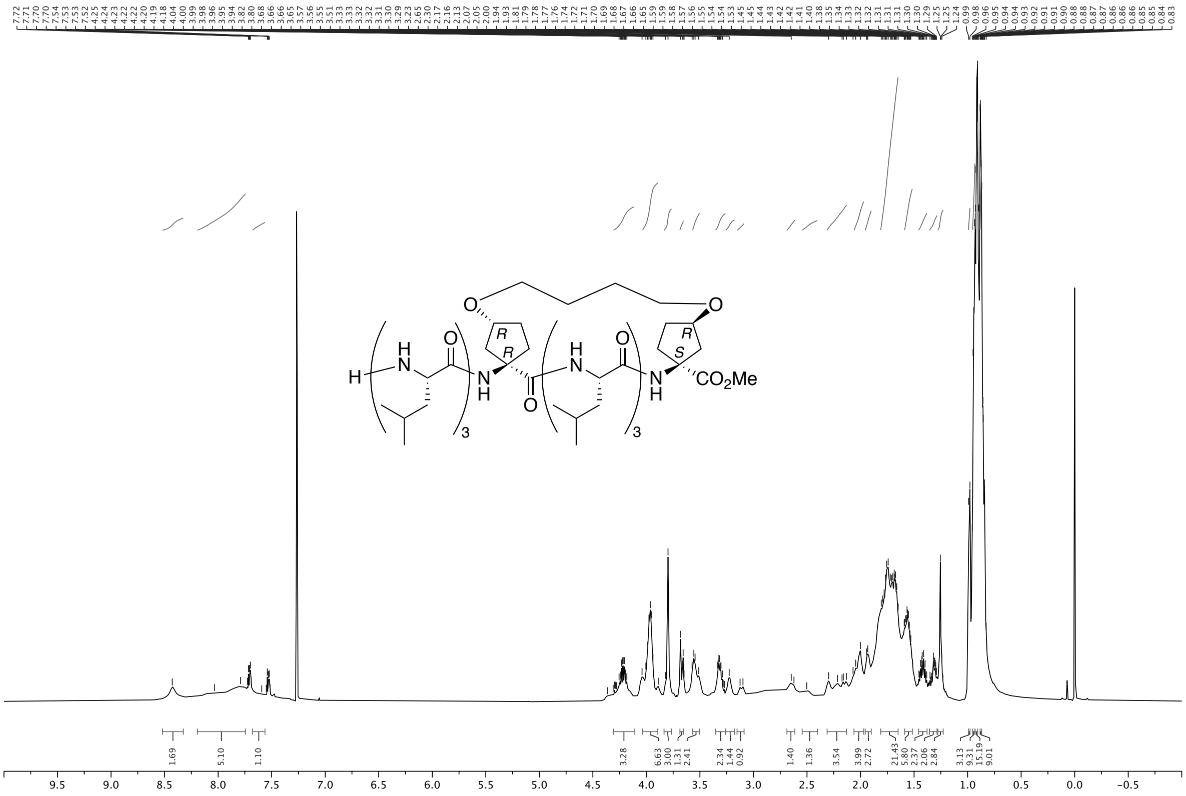

^{1}H NMR of **2e′** (500 MHz, $CDCl_3$)

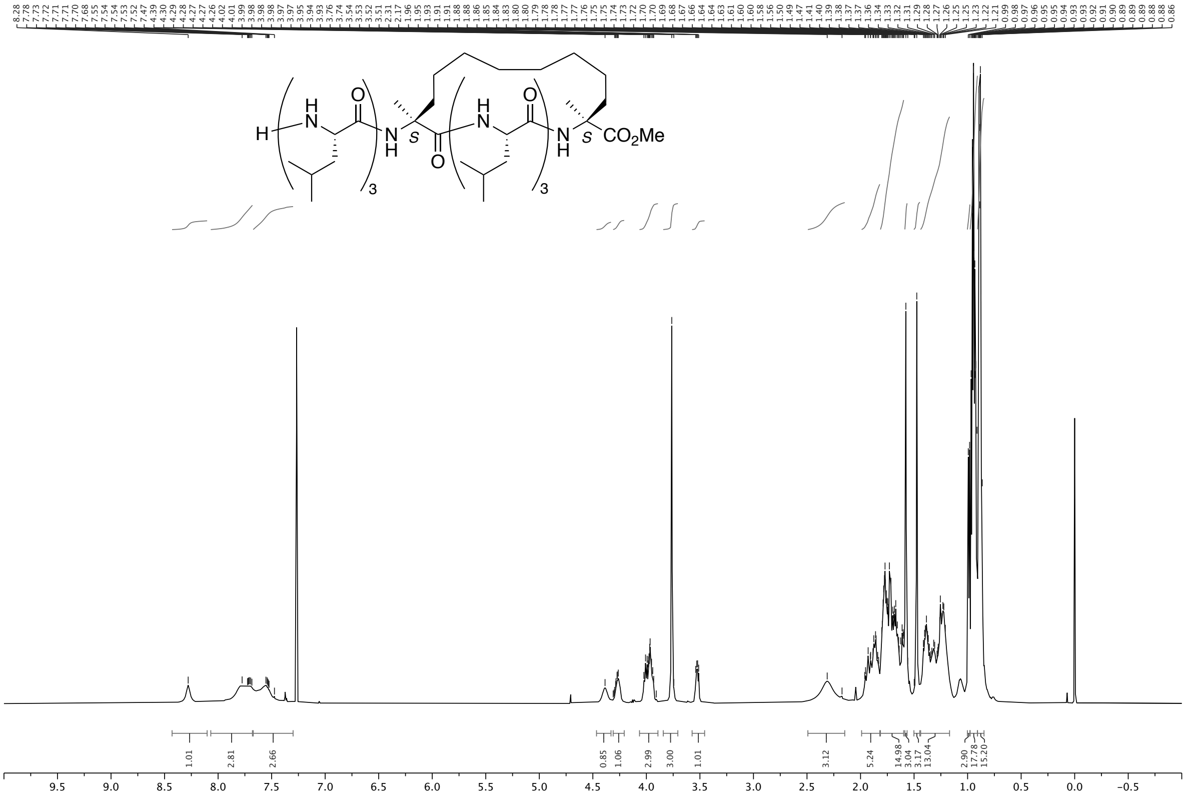

^{1}H NMR of **4a** (500 MHz, $CDCl_3$)

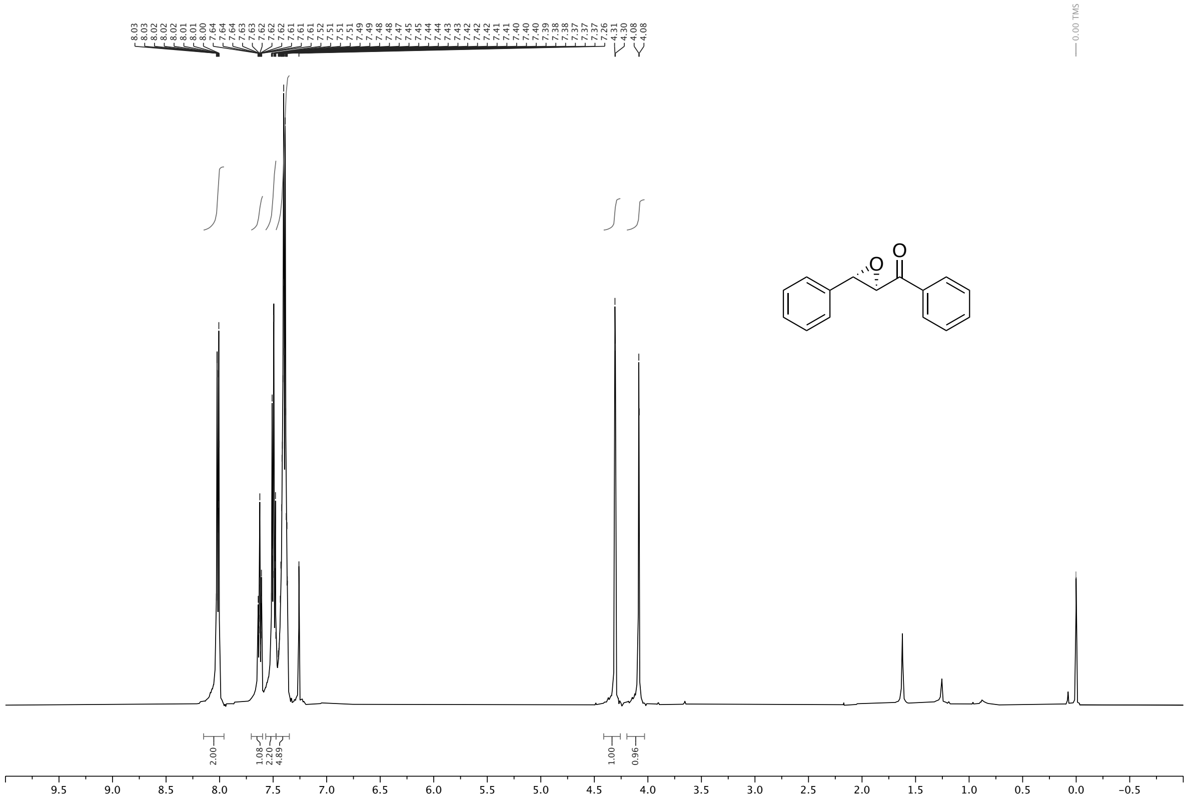

^{1}H NMR of **4b** (500 MHz, $CDCl_3$)

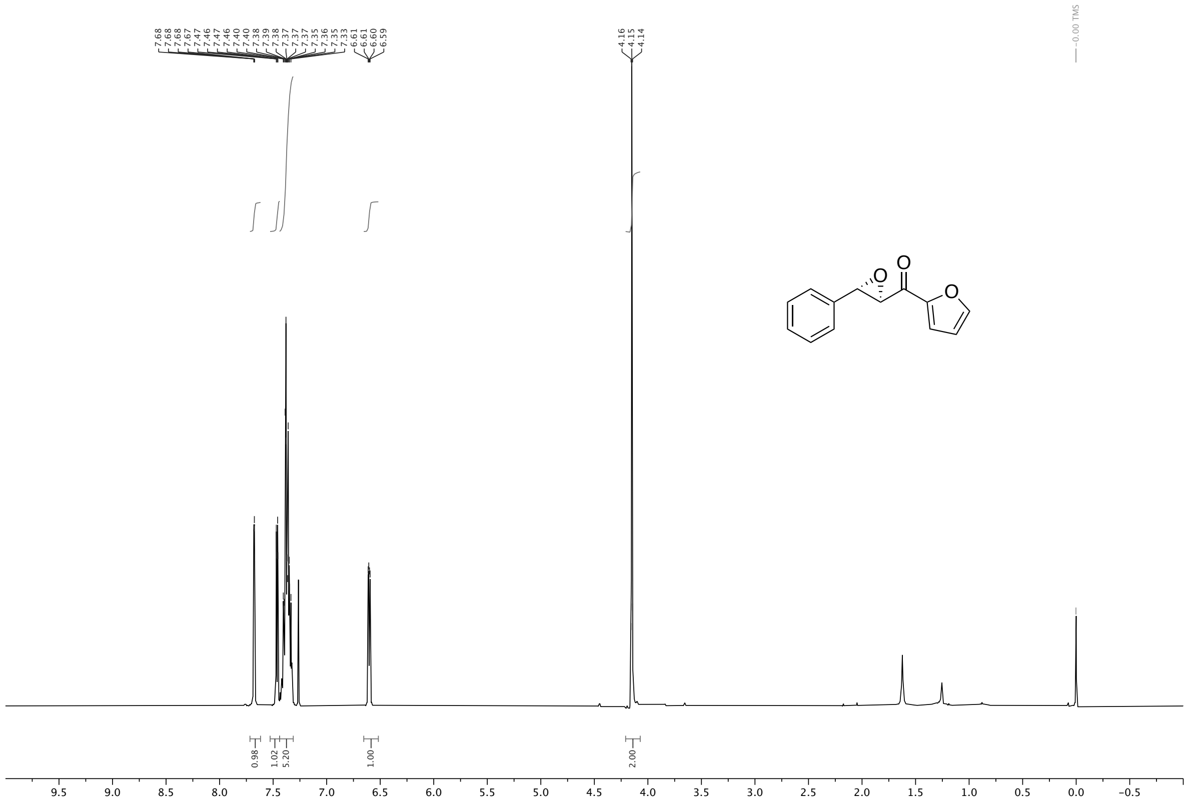

^{1}H NMR of **4c** (500 MHz, $CDCl_3$)

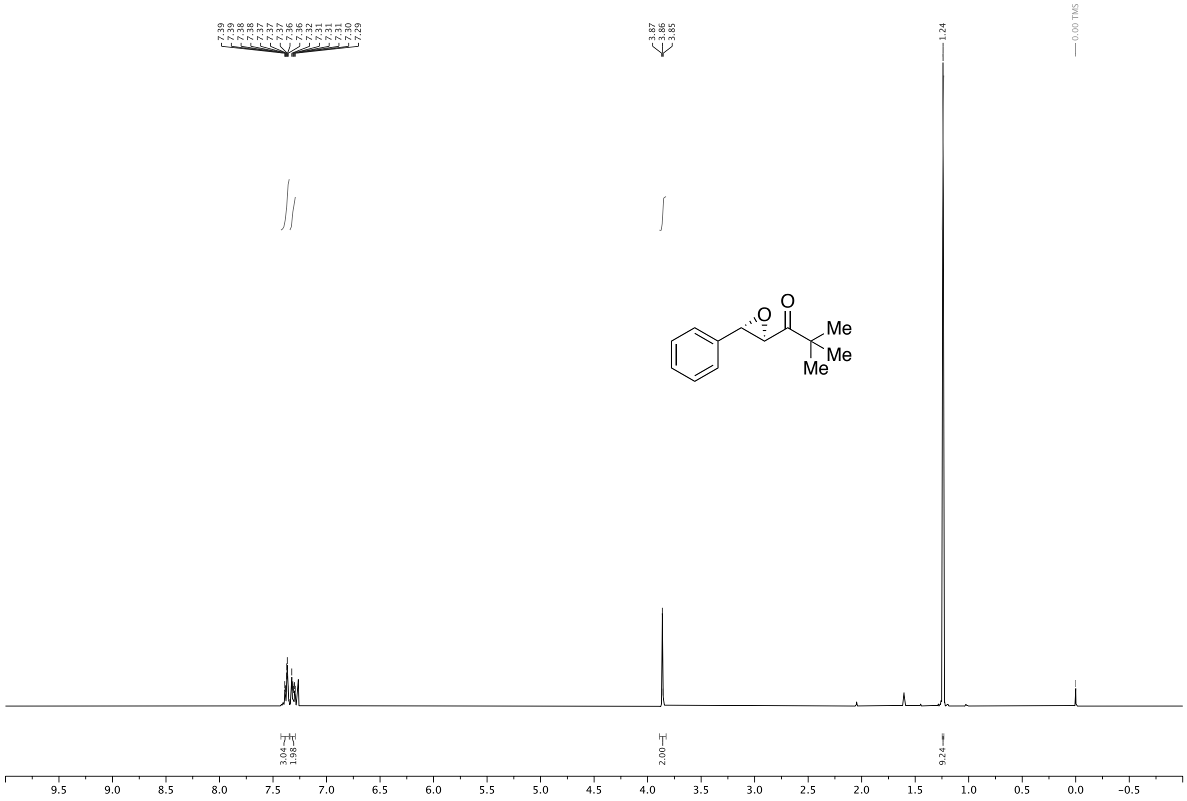

^{1}H NMR of **4d** (300 MHz, $CDCl_3$)

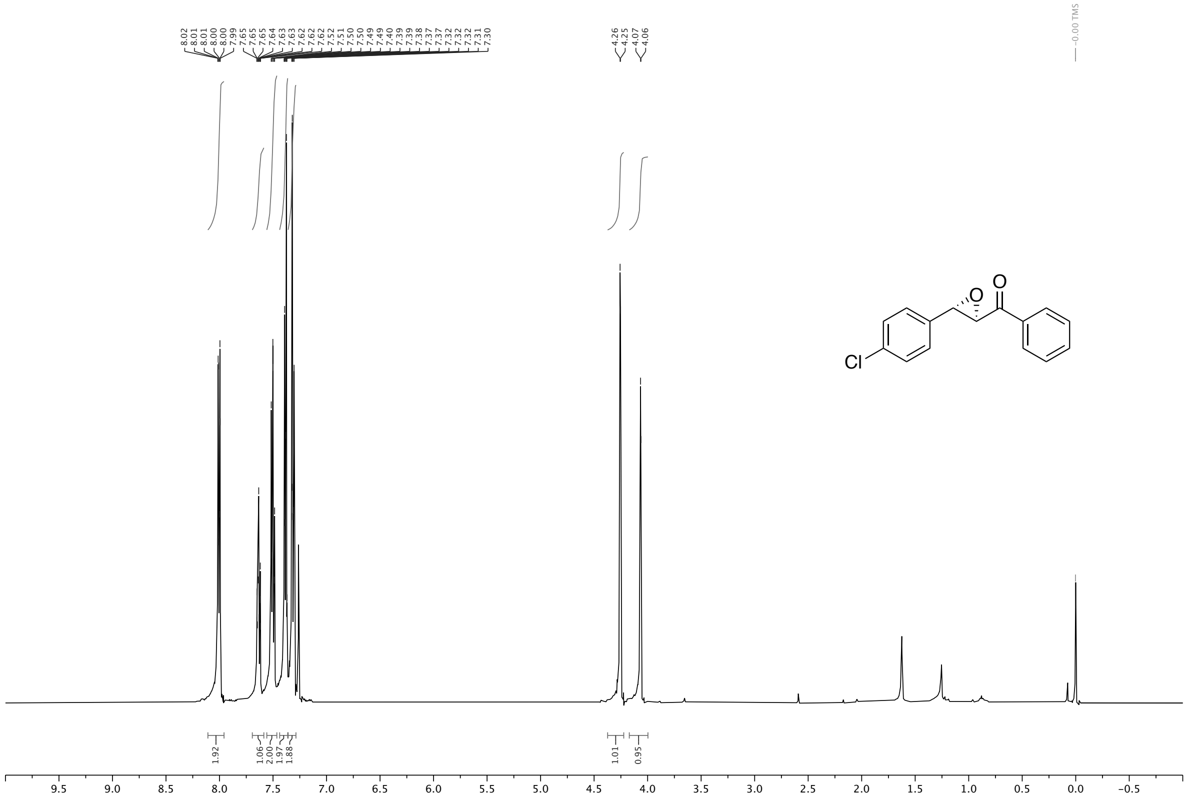

^{1}H NMR of **4e** (500 MHz, $CDCl_3$)

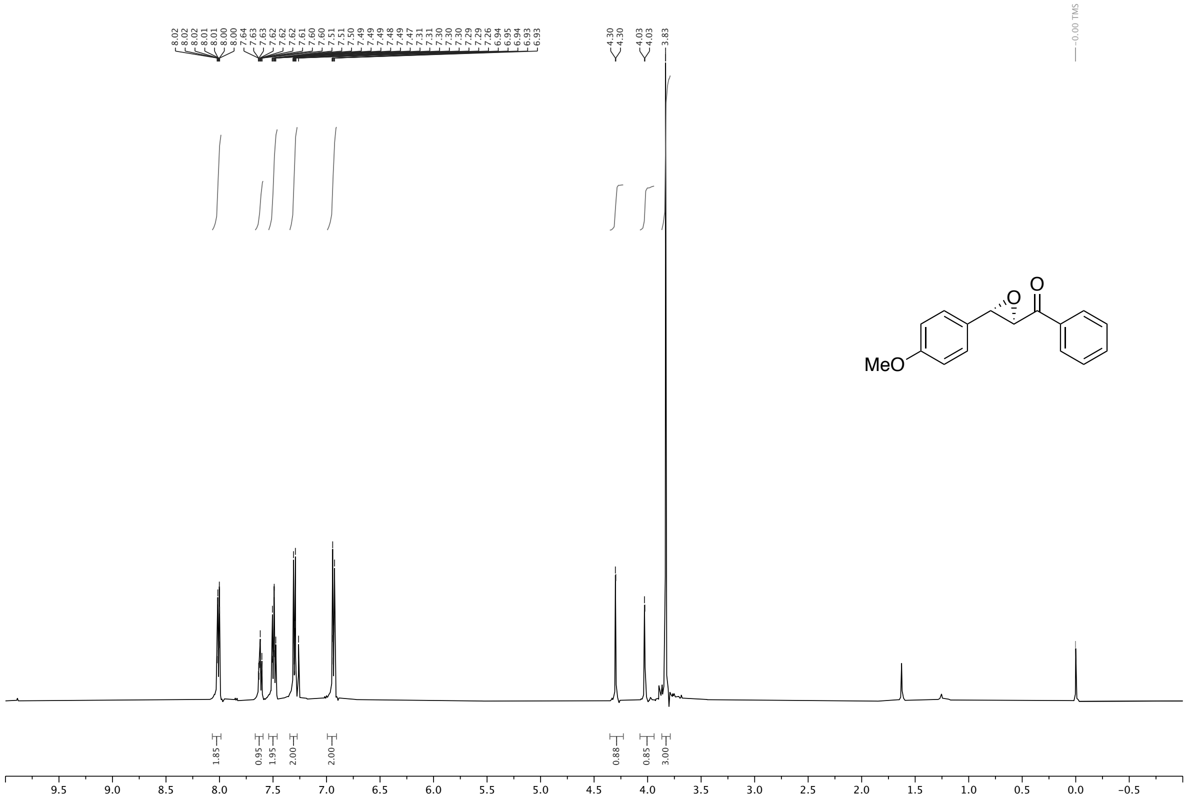

4. HPLC charts (top: racemic, bottom: peptide catalysis)

(2*R*,3*S*)-*trans*-Epoxy-3-phenyl-1-phenylpropan-1-one (4a)

Conditions: Chiralpak AD-H, 10% EtOH in *n*-hexane, flow rate = 1.0 mL/min.

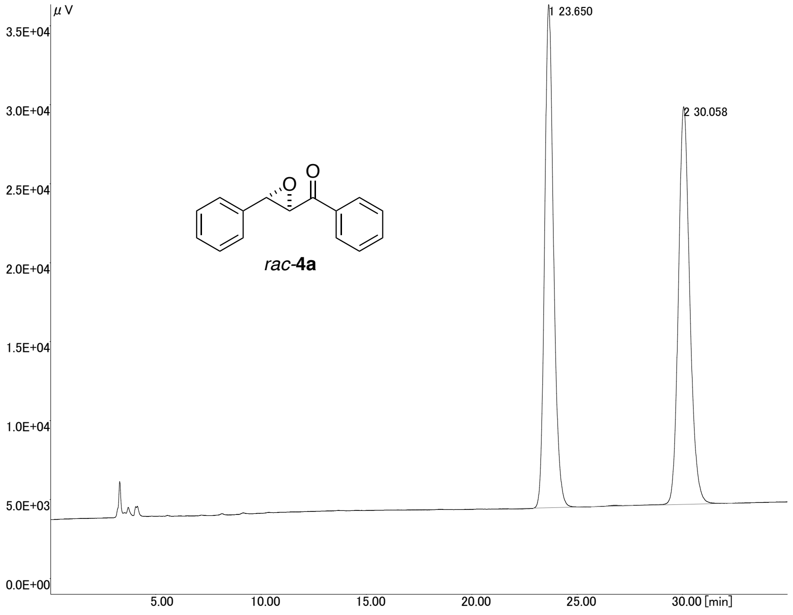

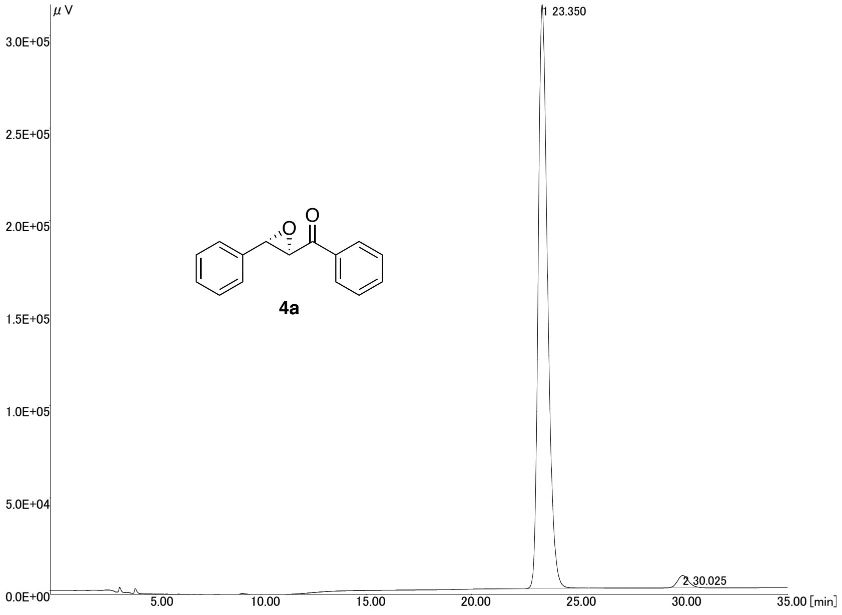

	Retention Time	Area	Area (%)
1	23.4	9831678.1	97.8
2	30.0	222139.1	2.2
Total		10053817.2	100

(2*R*,3*S*)-*trans*-Epoxy-3-phenyl-1-(2-furyl)propan-1-one (4b)

Conditions: Chiralpak IB N-5, 5% EtOH in *n*-hexane, flow rate = 1.0 mL/min.

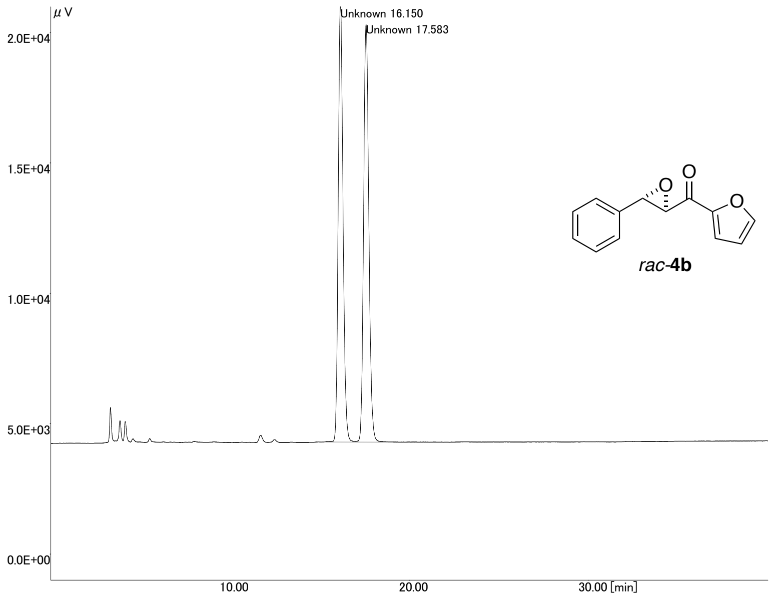

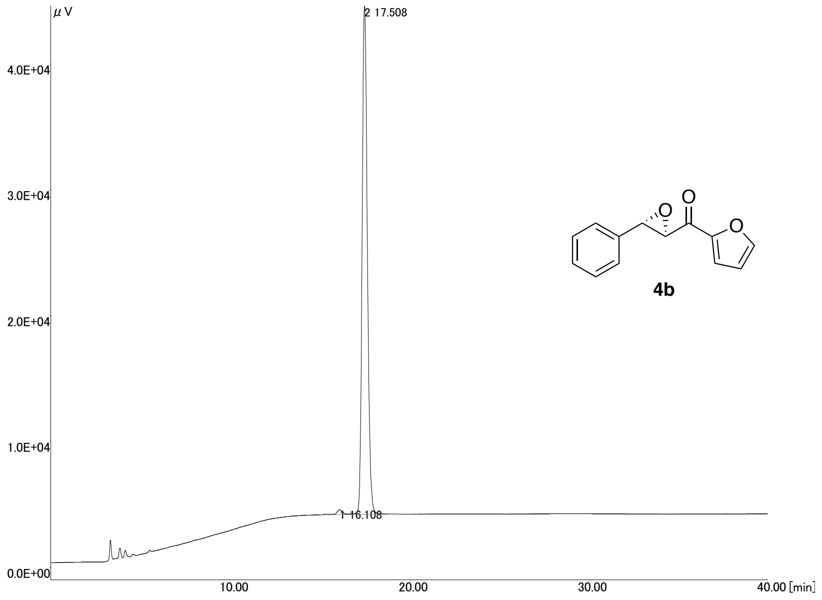

	Retention Time	Area	Area (%)
1	16.1	7843.2	1.00
2	17.5	796986.4	99.0
Total		804829.6	100

(1*S*,2*R*)-*trans*-1,2-Epoxy-4,4-dimethyl-1-phenylpentan-3-one (4c)

Conditions: Chiralpak AD-H, 5% EtOH in *n*-hexane, flow rate = 0.7 mL/min.

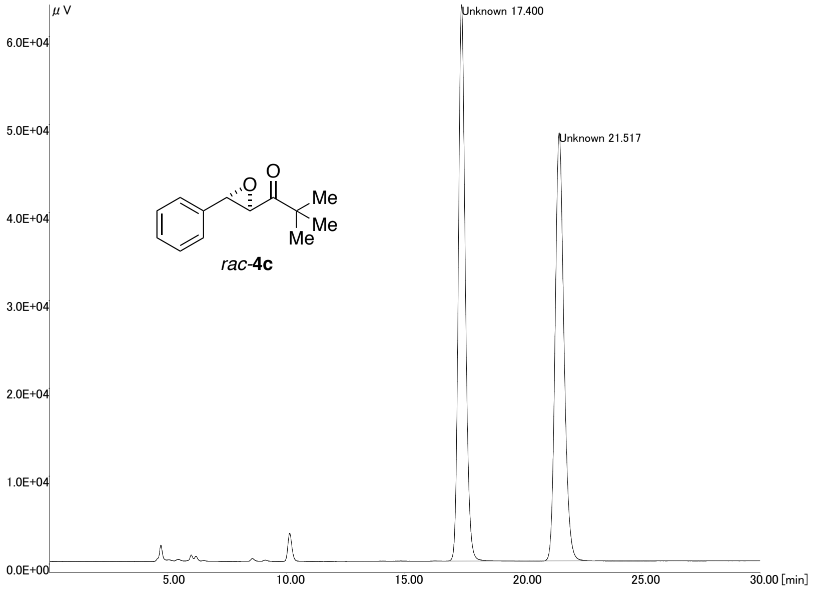

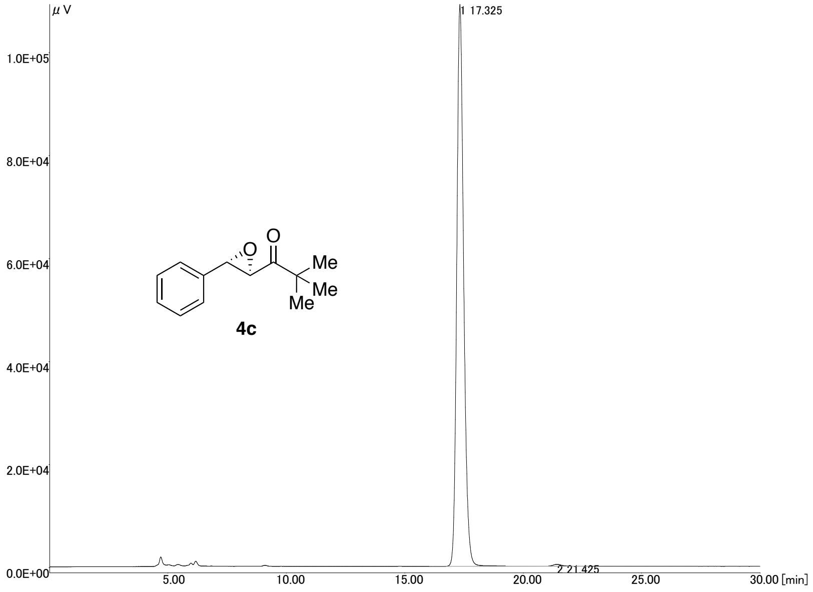

	Retention Time	Area	Area (%)
1	17.3	2198671.7	99.6
2	21.4	8903.4	0.4
Total		2189768.3	100

(2*R*,3*S*)-*trans*-Epoxy-3-(4-chlorophenyl)-1-phenylpropan-1-one (4d)

Conditions: Chiralpak IB N-5, 2% *i*-propanol in *n*-hexane, flow rate = 1.0 mL/min.

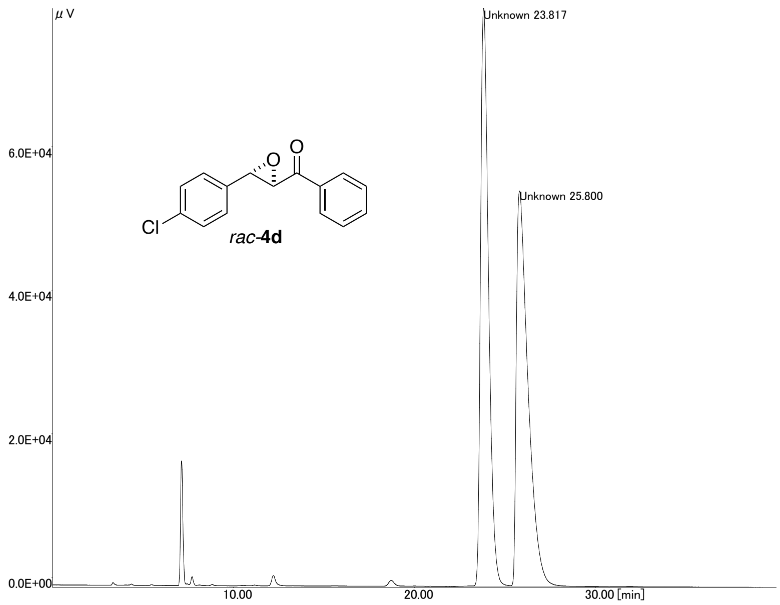

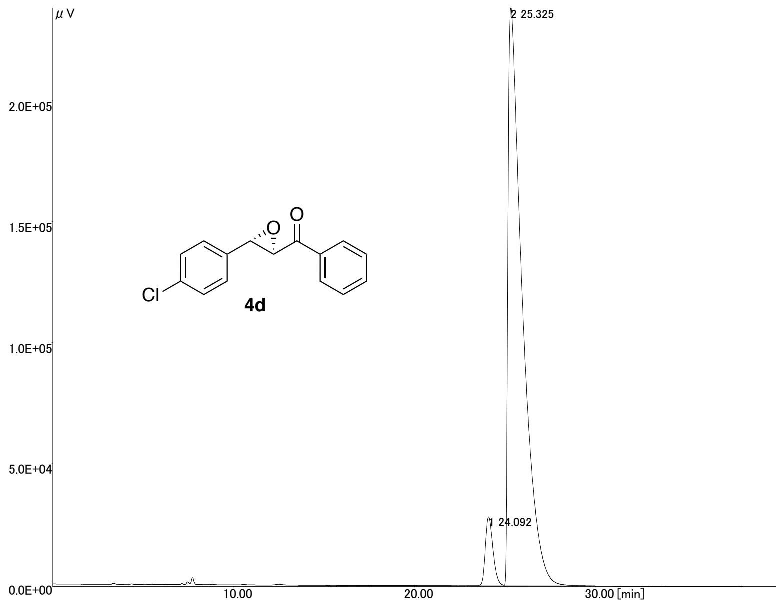

	Retention Time	Area	Area (%)
1	24.1	821147.6	5.9
2	25.3	13078308.7	94.1
Total		13899456.3	100

(2*R*,3*S*)-*trans*-Epoxy-3-(4-methoxyphenyl)-1-phenylpropan-1-one (4e)

Conditions: Chiralpak IB N-5, 2% *i*-propanol in *n*-hexane, flow rate = 1.0 mL/min.

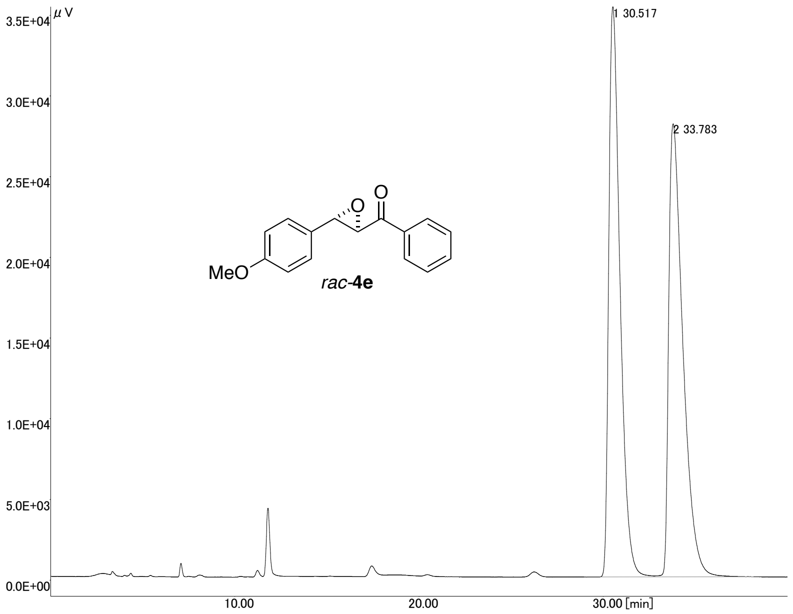

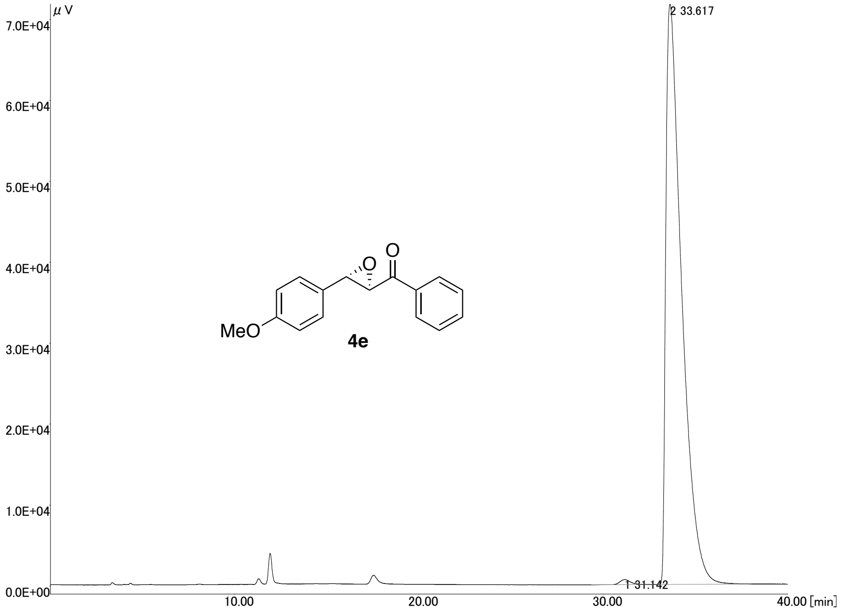

	Retention Time	Area	Area (%)
1	31.1	23795.5	0.6
2	33.6	4252181.7	99.4
Total		4275977.1	100
